# Genotoxic aldehyde stress prematurely ages hematopoietic stem cells in a p53-driven manner

**DOI:** 10.1016/j.molcel.2023.05.035

**Published:** 2023-06-21

**Authors:** Meng Wang, Laura T.L. Brandt, Xiaonan Wang, Holly Russell, Emily Mitchell, Ashley N. Kamimae-Lanning, Jill M. Brown, Felix A. Dingler, Juan I. Garaycoechea, Tomoya Isobe, Sarah J. Kinston, Muxin Gu, George S. Vassiliou, Nicola K. Wilson, Berthold Göttgens, Ketan J. Patel

**Affiliations:** 1 Division of Nutritional Sciences, Cornell University, Ithaca, NY, USA; 2 Wellcome-MRC Cambridge Stem Cell Institute, Jeffrey Cheah Biomedical Centre, University of Cambridge, Cambridge, UK; 3 MRC Laboratory of Molecular Biology, Francis Crick Avenue, Cambridge, UK; 4 School of Public Health, Shanghai Jiaotong University School of Medicine, Shanghai, China; 5 Wellcome Sanger Institute, Hinxton, UK; 6 Hubrecht Institute-KNAW (Royal Netherlands Academy of Arts and Sciences) and University Medical Center, Utrecht, the Netherlands; 7 MRC Weatherall Institute of Molecular Medicine, University of Oxford, John Radcliffe Hospital, Oxford, UK

## Abstract

Aged hematopoietic stem cells (HSCs) display diminished self-renewal and a myeloid differentiation bias. However, the drivers and mechanisms that underpin this fundamental switch are not understood. HSCs produce genotoxic formaldehyde that requires protection by the detoxification enzymes ALDH2 and ADH5 and the Fanconi anemia (FA) DNA repair pathway. We find that the HSCs in young *Aldh2^−/−^* Fancd2 ^−/−^ mice harbor a transcriptomic signature equivalent to aged wild-type HSCs, along with increased epigenetic age, telomere attrition, and myeloid-biased differentiation quantified by single HSC transplantation. In addition, the p53 response is vigorously activated in *Aldh2 ^−/−^* *Fancd2 ^−/−^* HSCs, while p53 deletion rescued this aged HSC phenotype. To further define the origins of the myeloid differentiation bias, we use a GFP genetic reporter to find a striking enrichment of Vwf+ myeloid and megakaryocyte-lineage-biased HSCs. These results indicate that metabolism-derived formaldehyde-DNA damage stimulates the p53 response in HSCs to drive accelerated aging.

## Graphical abstract

**Graphic abstract:**
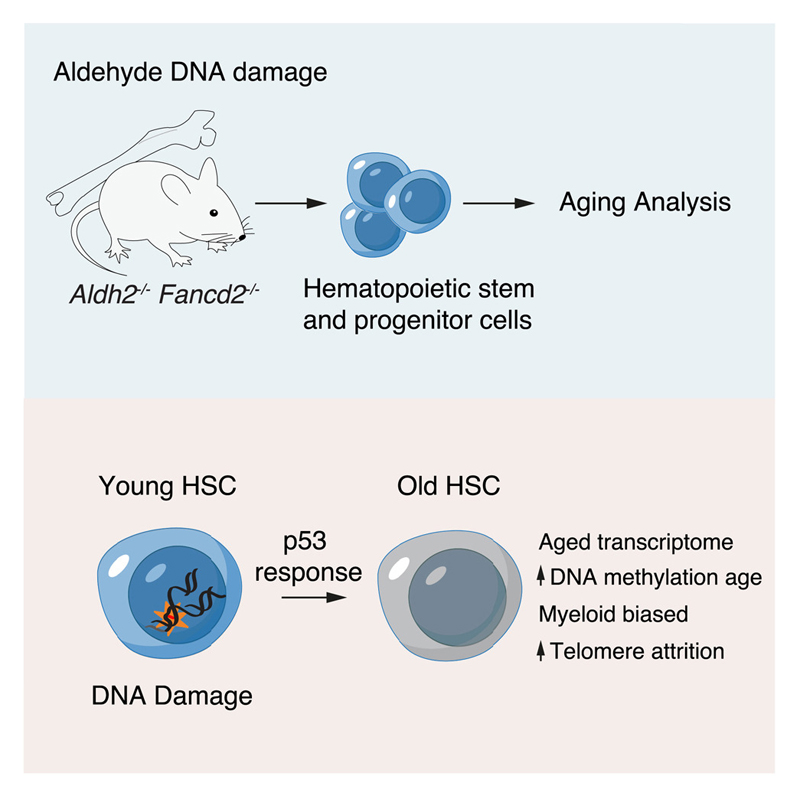

## Introduction

As we age, our blood changes. These changes are a consequence of alterations in hematopoietic stem cells (HSCs) that reside at the apex of blood production and have fundamental implications for our ability to replenish blood. Aged HSCs lose regenerative potential and shift their production of white blood cells to favor more myeloid cells and fewer lymphoid cells.^1–5^ In addition, mutated HSC clones begin to dominate blood production by outcompeting other cells in the HSC pool (clonal hematopoiesis).^6–11^ Ultimately, these mutated HSCs can degenerate to myelodysplastic syndromes and leukemia.^10,12,13^ However, the physiological processes that drive age-related HSC dysfunction are poorly understood. Mechanistic understanding of HSC aging might enable us to mitigate the degradation of blood production with profound medical benefits.

DNA damage has been proposed as a driver of HSC aging. Many of the properties seen in aged blood are frequently associated with somatic mutations in a small number of key genes commonly involved in genome stability *(TP53* and *PPM1D*), epigenetic and transcriptional regulators *(TET2, DNMT3A*, and *AXSL1*), and splicing (*SRSF2* and SF3B1).^6–9^ This correlation suggests that DNA damage and mutations can alter HSC output, increase clonal competitiveness, and drive neoplastic transformation. However, mutations of these driver genes are not universal or necessary for the aged blood phenotype.^1,2,4,14–16^ Thus, HSC aging cannot be solely explained by the acquisition of DNA mutations in HSCs.

As well as a source of mutations, DNA damage also induces a potent DNA-damage response (DDR) that negatively impacts HSCs. This is most strikingly observed in inherited deficiencies of genome maintenance, e.g., dyskeratosis congenita due to loss of telomere protection,^17^ and Fanconi anemia (FA) from lack of DNA crosslink repair.^18–20^ Children with these conditions suffer HSC attrition due to the DDR triggering the p53 response, which lead to apoptosis and senescence in HSCs and resultant failure of blood production from an early age.^21–23^ While the loss of HSCs by apoptosis is an extreme outcome of DDR activation, what is less clear is how and whether continued exposure to endogenous DNA damage and DDR can promote HSC aging.

A challenge in addressing the role of DDR in aging has been to identify and apply physiological DNA damage to recapitulate the changes observed in blood aging. Mice deficient in DNA repair pathways exhibit variable HSC impairments, but the endogenous physiological source of DNA damage in these models remains largely unknown.^24–29^ Endogenous factors that promote DNA damage in HSCs include fundamental cellular processes such as transcription and replication.^28,30^ In addition, the genome is under constant chemical attack from water and oxygen.^30,31^ All these factors are essential for cell survival, and thus difficult to study without compromising HSC fitness. We have recently identified formaldehyde as a ubiquitous chemical source of DNA damage in HSCs.^32,33^ Mammals produce high levels of this genotoxic aldehyde, requiring detoxification through two tiers of protection: the enzymes ALDH2 and ADH5 (tier 1), and DNA repair by the FA pathway (tier 2).^32–36^ Mice that lack both enzymatic detoxification and DNA repair accumulate DNA damage in HSCs, leading to their mutagenesis, attrition, and loss of function.^37^ Furthermore, in humans, congenital deficiency both in ALDH2 and ADH5 with intact DNA repair leads to an inherited bone marrow failure and leukemia syndrome similar to FA,^32,38,39^ indicating that endogenous formaldehyde exposure is a major physiological source of DNA damage in HSCs.

This study utilizes mice deficient in aldehyde detoxification to examine in detail how HSCs are altered by endogenous DNA damage caused by aldehydes. We uncover that in the absence of protection against this damage, murine HSCs mirror transcriptional and functional properties that are associated with aged HSCs. These changes are dependent on p53, and genetic deletion of this key DDR protein is sufficient to rejuvenate HSCs transcriptionally and functionally.

## Results

### Profiling transcriptomes of aldehyde stressed HSPCs by scRNA-seq

**Figure 1:**
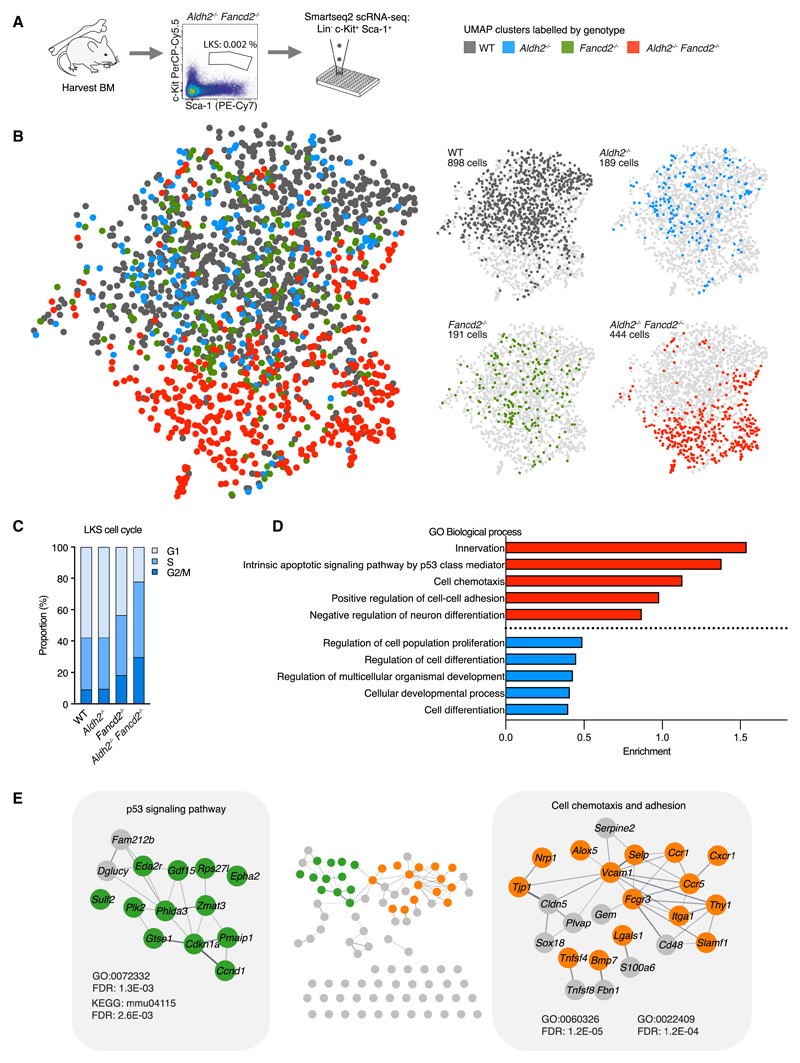
Single-cell transcriptomes of *Aldh2^−/−^ Fancd2^−/−^* stem and progenitor cells (A) scRNA-seq of LKS cells from 8- to 12-week-old *Aldh2^−/−^ Fancd2^−/−^* mice with age matched WT, *Aldh2^−/−^*, and *Fancd2^−/−^* controls. See also Figure S1. (B) Uniform manifold approximation and projection (UMAP) visualization of LKS transcriptomes colored by genotype with UMAP of all 4 genotypes superimposed on the left, and individual genotypes on the right. (C) Proportion of LKS cells in different cell-cycle phases as determined by transcriptome profile. (D) GO terms ranked by gene enrichment from top 100 upregulated (red) and downregulated genes (blue) in LKS from *Aldh2^−/−^ Fancd2^−/−^* compared with WT (FDR < 0.01, redundant GO terms omitted). See also Tables S1 and S2. (E) String network of top 100 upregulated genes described in (D). visualized in cytoscape V3.9.0, with gene nodes highlighted from the respective GO and Kyoto Encyclopedia of Genes and Genomes (KEGG) terms.

We have previously shown that deficiency in ALDH2 or ADH5 combined with deletion of the FA pathway results in loss of HSCs and a lethal failure of hematopoiesis in mice.^33,34^ *Adh5^−/−^ Fancd2^−/−^* mice suffer severe loss of blood production from an early age resulting in perinatal lethality (median 33 days). In contrast, *Aldh2^−/−^ Fancd2^−/−^* mice survive longer (median 145 days) and develop anemia and cytopenia beyond 10 weeks of age. We therefore set out to study hematopoietic stem and progenitor cells (HSPCs) in 8- to 12-week-old *Aldh2^−/−^ Fancd2^−/−^* mice to capture how endogenous genotoxic aldehydes impact and reshape hematopoiesis. The majority of *Aldh2^−/−^ Fancd2^−/−^* mice at this age exhibit significantly reduced number of Lineage^−^ [Lin^−^] c-Kit^+^ Sca-1^+^ (LKS) HSPCs (Figure S1) but can maintain sufficient blood production to prevent symptomatic anemia. To interrogate and dissect the HSPC population, we subjected LKS HSPCs to single-cell RNA sequencing (scRNA-seq) using the plate-based Smartseq2 platform^40^ (Figures 1A and 1B). The *Aldh2^−/−^ Fancd2^−/−^* HSPC transcriptomes show a greater proportion of S and G2/M cycling cells (Figure 1C), in agreement with our prior observations.^34^ Regressing out these cell-cycle phase variations did not eliminate the striking differences between HSPCs from *Aldh2^−/−^ Fancd2^−/−^* compared with other genotypes (Figure 1B). To gain insight into the differentially expressed genes that underpin the molecular differences in *Aldh2^−/−^ Fancd2^−/−^* HSPCs, we applied gene ontology analysis to find enrichment of gene ontology (GO) terms associated with the *Trp53* signaling pathway (*Cdkn1a*, *Pmaip1*, *Ccnd1*, *Gtse1*, *Phlda3*, *Zmat3*, *Plk2*, *Sulf2*, *Epha2*, *Rps27l*, *Gdf15*, and *Eda2r) in Aldh2^−/−^ Fancd2^−/−^* HSPCs (Figures 1D and 1E; Tables S1 and S2). This is consistent with our previous findings,^32,37^ demonstrating an activated DDR in HSPCs under genotoxic aldehyde stress. In addition, we find that *Aldh2^−/−^Fancd2^−/−^* HSPCs exhibit differentially expressed genes associated with cell adhesion (*Selp*, *Itga1*, *Vcam1*, and *Lgals1*), cell chemotaxis (*Ccr1*, *Cxcr1*, and *Ccr5*) and differentiation (Mpl and Flt3). We also find GO terms associated with innervation and neuron differentiation, which supports other reports of common pathways involved in regulation of both hematopoietic and neuronal stem cells.^41–43^ In summary, the transcriptomes of *Aldh2^−/−^ Fancd2^−/−^* HSPCs show significantly increased p53 activity, upregulation of cell membrane genes involved in chemotaxis and adhesion, and differentiation-related genes.

### Two-tier-deficient HSCs transcriptionally mirror aged HSCs

The significant upregulation of genes encoding membrane proteins in *Aldh2^−/−^ Fancd2^−/−^* HSPCs was unexpected. While several of these genes can be upregulated in response to cellular stimulation and stress,^44,45^ we were intrigued by the association between the these membrane protein genes and HSC aging in mice.^46^ This prompted us to assess whether the HSCs from 8-to 12-week-old *Aldh2^−/−^ Fancd2^−/−^* mice showed evidence of accelerated aging.

**Figure 2:**
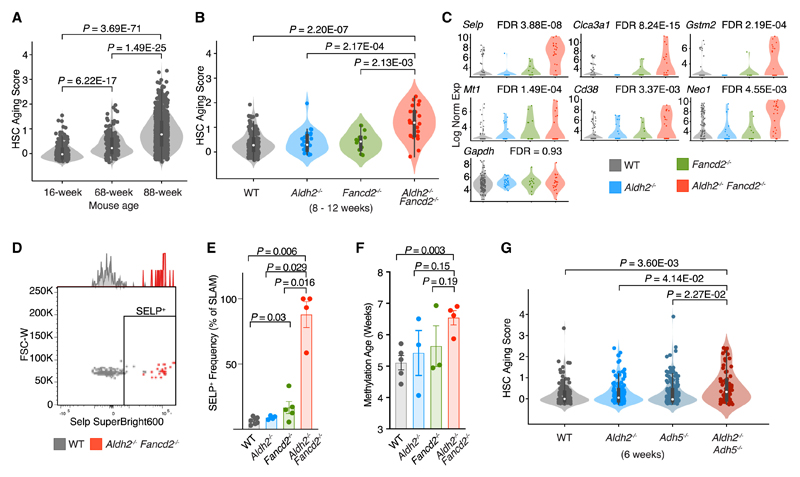
*Aldh2^−/−^ Fancd2^−/−^* HSPCs exhibit increased aging (A and B) Aging score analysis of LKS transcriptomes identified as LT-HSCs in (A). 16- and 68-week-old WT mice, and an 88-week-old *Fancd2^+/−^* mouse (n = 234, 187, and 758 left to right), (B) 8- to 12-week-old *Aldh2^−/−^ Fancd2^−/−^* and control mice (n = 162, 27, 14, and 23 left to right). (C) Expression of aging-associated genes in LT-HSCs. FDR represents comparison between *Aldh2^−/−^ Fancd2^−/−^* and WT. (D and E) SELP expression in SLAM HSCs (Lin^−^ Kit^+^ Sca-1^+^ CD48^−^ CD150^+^) shown in representative flow cytometry (D) and bar plot quantification (mean ± SEM; n = 7, 4, 5, and 4, left to right) (E). (F) DNA methylation age of bone marrow cells from 10-week-old mice (mean ± SEM; n = 5, 3, 3, and 4, left to right). (G) Aging score analysis of LT-HSC scRNA-seq transcriptomes from 6-week-old *Aldh2^−/−^ Adh5^−/−^* and control mice (n = 172, 166, 184, and 77 left to right).

We initially utilized a transcriptome-based HSC aging signature (AS),^46^ consisting of 20 genes that were most consistently reported to be differentially expressed between aged (2 years) and young (8 weeks) murine long-term HSCs (LT-HSCs). We validated this AS in scRNA-seq transcriptomes of LT-HSCs from a young (16 weeks) and a mid-aged (68 weeks) wild-type (WT) mouse, and an old (88 weeks) *Fancd2^+/–^* mouse; where *Fancd2* heterozygosity is not known to confer any phenotype compared with WT mice. This reveals heterogeneity among the AS score distribution of LT-HSCs, but the overall AS score progressively increases with the chronological age of the LT-HSCs, thus demonstrating that the transcriptome-based AS score correlates with HSC age (Figure 2A). We then applied the AS score to LT-HSCs from 8- to 12-week-old *Aldh2^−/−^ Fancd2^−/−^* mice, which exhibit significantly increased AS score compared with LT-HSCs from WT and single knockout control mice (Figure 2B). Interestingly, the *Aldh2^−/−^ Fancd2^−/−^* LT-HSCs display similar AS score heterogeneity as the LT-HSCs from the 68- and 88-week-old mice and suggests that the biological age of individual HSCs vary within a population of similar chronological age. In addition, we observe upregulation of several genes whose expression were consistently correlated to LT-HSC aging (*Selp*, *Mt1*, *Gstm2*, *Clca3a1*, *Cd38*, and *Neo1*) (Figure 2C). Furthermore, we immunophenotypically validated the upregulation of the top aging marker gene *Selp*, encoding for the P-selectin surface protein in *Aldh2^−/−^ Fancd2^−/−^* SLAM LT-HSCs (Lin^–^ c-Kit^+^ Sca-1^+^ CD48^–^ CD150^+^)(Figures 2D and 2E). Aging-associated changes in DNA methylation has been widely adopted as an epigenetic clock that can be predictive of age acceleration.^47^ We assessed the DNA methylation age of hematopoietic cells from the bone marrow to find increased methylation age in *Aldh2^−/−^ Fancd2^−/−^* compared with WT mice (Figure 2F) and is consistent with observations from other DNA damage murine models that exhibit accelerated epigenetic aging in blood.^48,49^

While *Aldh2^−/−^ Fancd2^−/−^* mice lack both aldehyde detoxification and DNA repair, we have also discovered that mice deficient in aldehyde detoxification enzymes ALDH2 and ADH5 accumulates elevated endogenous formaldehyde-DNA damage despite intact DNA repair response.^32^ *Aldh2^−/−^ Adh5^−/−^* mice suffer loss of HSCs, but the depletion of HSCs is not as severe as that observed in *Aldh2^−/−^ Fancd2^−/−^* mice. Humans born with inherited mutations in both ALDH2 and ADH5 develop early bone marrow failure and leukemia akin to that observed in FA.^32,38^ We therefore asked whether a similar premature aging of HSCs could be present in *Aldh2^−/−^ Adh5^−/−^* mice. Upon re-analysis of our published scRNA-seq dataset of HSPCs from *Aldh2^−/−^ Adh5^−/−^* mice, we find an increased AS score in the *Aldh2^−/−^ Adh5^−/−^* LT-HSCs compared with WT and single knockout LT-HSCs. This result indicates that an orthogonal model of formaldehyde-driven endogenous DNA damage resembles the aged transcriptome in *Aldh2^−/−^ Fancd2^−/−^* HSCs (Figure 2G).

### Myeloid progenitors dominate the two-tier-deficient HSPC transcriptomic landscape

**Figure 3:**
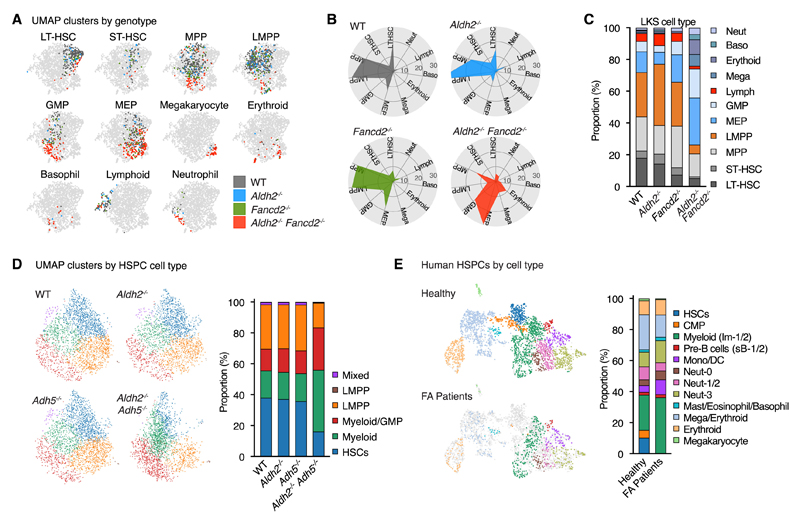
Aldehyde-sensitive murine and human FA HSPCs exhibit myeloid bias (A-C) LKS cell types identified based on transcriptome identity shown by (A). UMAP distribution, (B) polar plot, and (C) bar plot showing each cell type as a proportion of total LKS cells. See also Figures S2 and S3. (D and E) UMAP visualization and bar plot quantification of each cell type as a proportion of total HSPCs based on transcriptome identity in *Aldh2^−/−^ Adh5^−/−^* and control mice (D), and healthy humans and FA patients (E).

Another property of aged hematopoiesis is increased differentiation bias to myeloid lineages.^1,2^ Therefore, we proceeded to identify myeloid- and lymphoid-primed HSPCs based on their transcriptome. By profiling marker genes and projecting single-cell transcriptomes onto reference datasets,^50,51^ we were able to distinguish LT-HSCs and short-term HSCs (ST-HSCs), as well as multipotent progenitors (MPPs), lympho-myeloid-primed progenitors (LMPPs), granulocyte/monocyte progenitors (GMPs), megakaryocyte-erythroid progenitors (MEPs), and progenitor cells committed to the megakaryocyte, erythroid, basophil, lymphoid, and neutrophil lineages (Figure 3A). GO term analysis in each cell type shows consistent enrichment of cell adhesion and migration GO terms in multiple *Aldh2^−/−^Fancd2^−/−^* HSPC cell types (Table S3). In HSPCs of *Aldh2^−/−^ Fancd2^−/−^*, there were reduced proportions of cells with the transcriptomic profiles of LT-HSCs, ST-HSCs, and MPPs, relative expansion of myeloid progenitors (GMPs, MEPs, megakaryocytes, erythroid, basophil, and neutrophil progenitors) and depletion of lymphoid progenitors (LMPP and lymphoid progenitors) (Figures 3B and 3C). We also observe similar myeloid bias in the HSPC scRNA-seq analysis of *Aldh2^−/−^ Adh5^−/−^* hematopoiesis (Figure 3D), and in a published human FA patient HSPC scRNA-seq dataset (Figure 3E).^52^

Systemic inflammation is a known driver of myeloid bias.^53–57^ However, we found comparable levels of pro-inflammatory cytokines between WT and *Aldh2^−/−^ Fancd2^−/−^* serum (Figure S2A). In addition, we do not observe obvious changes between WT and *Aldh2^−/−^ Fancd2^−/−^* HSPCs in the expression of lipopolysaccharide-induced inflammatory response genes^58^ (Figure S2B). In the absence of detectable inflammatory response, this suggests that inflammation cannot be the dominant driver of the myeloid bias in *Aldh2^−/−^ Fancd2^−/−^* mice. We do observe upregulation of transforming growth factor β (TGF-β) pathway in *Aldh2^−/−^ Fancd2^−/−^* HSPCs, in agreement with prior studies in FA deficient mice and humans^59,60^ (Figure S2C). Prior studies have observed *Batf* and *Per2* upregulation in HSCs following DNA damage to drive HSC differentiation and lymphoid depletion.^61,62^ In *Aldh2^−/−^ Fancd2^−/−^* mice, we do not observe differences in the expression of *Batf* or *Per2* in the transcriptomic profiles of LKS cells, LT-HSCs, or LMPPs compared with WT mice (Figure S3). In summary, our findings show that in the absence of aldehyde protection, HSCs exhibit aged transcriptomes, increased epigenetic age, and myeloid bias. These features are consistent with the accelerated aging of these cells.

### *p53* deletion reverses HSC aging and restores myeloid differentiation bias

**Figure 4:**
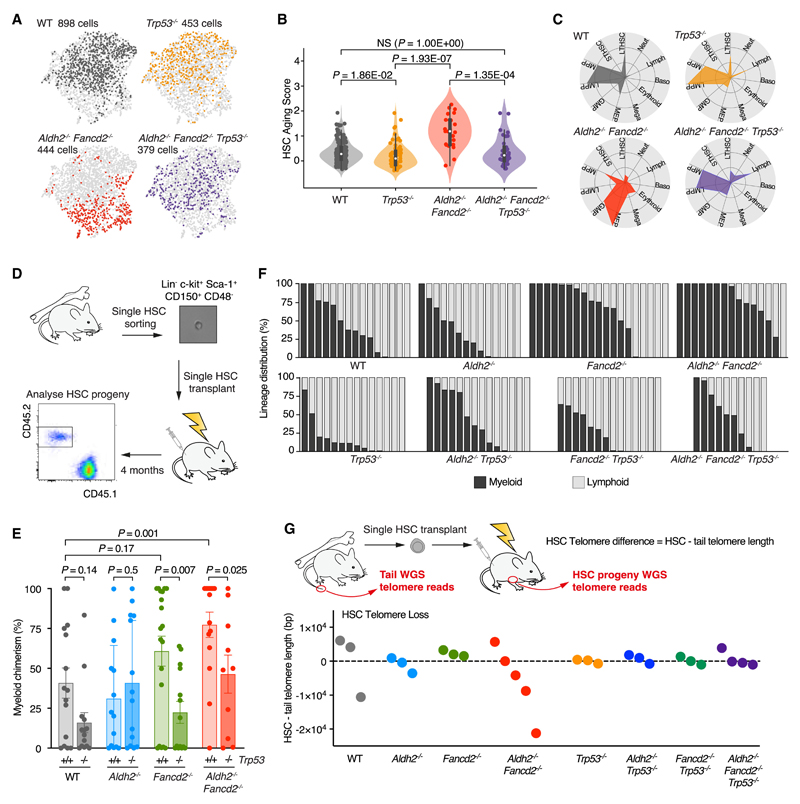
p53 drives HSC aging and myeloid bias in *Aldh2^−/−^ Fancd2^−/−^* mice (A) UMAP of LKS transcriptomes from *Trp53^−/−^* and *Aldh2^−/−^ Fancd2^−/−^ Trp53^−/−^* compared with WT and *Aldh2^−/−^ Fancd2^−/−^.* (B) Aging score of LT-HSC transcriptomes (n = 162, 71, 23, and 33, left to right), see also Figure S4A. (C) Polar plots showing the LKS cell lineage distribution, see also Figure S4B. (D) Scheme of single LT-HSC transplantation and analysis of myeloid or lymphoid progeny. (E) Myeloid chimerism defined as the proportion of myeloid progeny of singly transplanted HSCs (mean ± SEM; n = 15, 14, 14, 14, 19, 14, 15, and 10, left to right). (F) Lineage distribution showing the proportion of myeloid and lymphoid output from singly transplanted HSCs. Each column represents the output from a single HSC, see also Figures S4C and S4D. (G) Telomere length estimation derived from WGS of progenies of singly transplanted LT-HSCs and paired tail samples from the LT-HSC donor mouse. HSC telomere difference calculated by subtracting LT-HSC by the paired tail telomere length. Each point represents telomere difference of a singly transplanted LT-HSC.

As well as an aged transcriptomic signature, *Aldh2^−/−^Fancd2^−/−^*HSPCs display significant upregulation of p53 target genes. We have previously shown that the p53 response triggers HSC depletion and reconstitution defects in *Aldh2^−/−^Fancd2^−/−^* mice.^37^ Therefore, to elucidate the mechanism that drives accelerated aging in *Aldh2^−/−^ Fancd2^−/−^* HSPCs, we focused on the role of p53 transcriptional activity as a potential driver of HSC aging. We applied scRNA-seq analysis to LKS cells from *Trp53^−/−^* and *Aldh2^−/−^ Fancd2^−/−^ Trp53^−/−^* mice. In contrast to *Aldh2^−/−^ Fancd2^−/−^* mice, the *Aldh2^−/−^ Fancd2^−/−^ Trp53^−/−^* HSPC transcriptomes show greater similarity to that of WT HSPCs (Figure 4A). The AS score of *Aldh2^−/−^Fancd2^−/−^Trp53^−/−^* LT-HSCs is normalized back to be comparable to WT LT-HSCs (Figures 4B and S4A) and exhibit a more balanced proportion of myeloid- and lymphoid-primed HSPCs based on transcriptomic profiling (Figures 4C and S4B). Surprisingly, we find *Trp53^−/−^* LT-HSC transcriptomes harbor a lower AS score than WTLT-HSCs, thus implying a role for p53 in HSC aging in homeostatic hematopoiesis in WT mice. To functionally validate whether p53 underpins the myeloid differentiation bias in *Aldh2^−/−^Fancd2^−/−^* HSPCs and determine if the bias occurs at the most immature cell state within the hematopoietic hierarchy, we transplanted single LT-HSCs from *Aldh2^−/−^ Fancd2^−/−^*, *Aldh2^−/−^ Fancd2^−/−^ Trp53^−/−^*, and respective control mice (Figure 4D). After 4 months, we quantified the contribution to total blood cells (Figure S4C) and relative myeloid and lymphoid cells derived from the single engrafted HSC (Figures 4D–4F). Compared with singly transplanted HSCs from WT and *Aldh2^−/−^* mice, the singly transplanted HSCs from *Fancd2^−/−^* and *Aldh2^−/−^ Fancd2^−/−^* favored production of myeloid cells. Importantly, we did not observe HSC myeloid bias as an artifact of low HSC reconstitution (Figures S4C and S4D). Upon deletion of *Trp53^−/−^*, we found that the myeloid bias in *Fancd2^−/−^* and *Aldh2^−/−^ Fancd2^−/−^* HSCs was abolished, suggesting that a p53 response can induce the myeloid bias in HSCs that suffer DNA damage. To further investigate the role of p53 in HSC aging, we assessed telomere shortening in HSCs, which is another feature of aged cells. This was achieved computationally by applying the Telomerecat algorithm^63^ to reanalyze our previously published whole genome sequence (WGS) of *Aldh2^−/−^ Fancd2^−/−^* LT-HSCs.^37^ For each mouse, we estimated telomere difference in HSCs by subtracting the telomere length of LT-HSC by the paired tail sample (Figure 4G). Although the number of LT-HSCs analyzed was low, the *Aldh2^−/−^ Fancd2^−/−^* LT-HSCs clearly undergoes greater telomere loss compared with WT and single knockout LT-HSCs. Following deletion of the p53 response, *Aldh2^−/−^ Fancd2^−/−^Trp53^−/−^* mice harbored LT-HSCs with minimal telomere attrition (Figure 4G). In conclusion, the accelerated aging and myeloid bias in *Aldh2^−/−^ Fancd2^−/−^* HSCs is driven by p53 in response to endogenous DNA damage.

### Characterizing the p53 response in HSPCs

**Figure 5:**
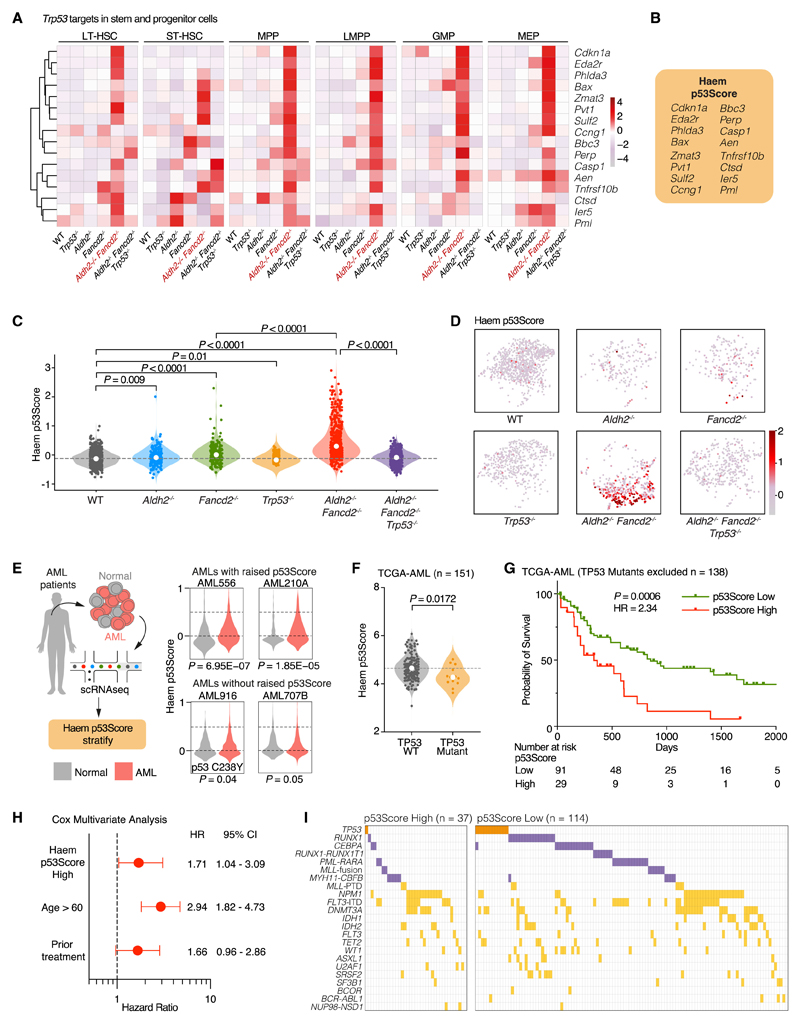
Haem p53Score to quantify p53 activation in normal and malignant hematopoiesis (A) Expression heatmap of p53 target genes upregulated in multiple HSPC populations of *Aldh2^−/−^ Fancd2^−/−^* compared with WT, and *Aldh2^−/−^ Fancd2^−/−^ Trp53^−/−^* mice. See also Figure S5A. (B) Haem p53Score quantifies *Trp53* activity in HSPCs based on the expression of 16 p53 target genes. (C and D) Haem p53Scores in LKS transcriptomes of each genotype quantified in violin plot (C) (median, n = 898, 189, 191, 453, 444, and 379, left to right), and visualized on UMAP (D). See also Figure S5B. (E) Interrogating human AML scRNA-seq dataset using the Haem p53Score reveals two patients (AML556 and AML210A) with elevated Haem p53Score in AML cells compared with normal cells, and two patients (AML916 and AML707B) with Haem p53Score in AML cells comparable to normal cells. AML916 harbors the *TP53* variant C238Y. (F) Haem p53Score analysis AMLs with WT and mutated *TP53* from the TCGA-AML database. (G) Kaplan Meier (KM) analysis showing probability of survival of *TP53* wild-type AML cases stratified by high (top 25%) or low (bottom 75%) p53Score (n = 138 TP53 WT AMLs), HR, hazard ratio of increased risk of death in p53Score high AMLs. (H) Multivariate Cox regression analysis of high Haem p53Score, aged >60 and prior treatment as independent predictors of increased mortality. See also Figure S6 and Table S4. (I) The spectrum of driver mutations found in AMLs with high and low Haem p53Score. *TP53* mutation highlighted in orange, class II mutations that inhibit AML differentiation highlighted in purple, other driver mutations highlighted in yellow. See also Table S5.

We set out to characterize the hematopoietic p53 transcriptional network in more detail. We compared the expression profiles of 113 validated p53 transcriptional targets^64^ in HSPC subsets (LT-HSCs, ST-HSCs, MPPs, LMPPs, GMPs and MEPs) from *Aldh2^−/−^Fancd2^−/−^, Aldh2^−/−^ Fancd2^−/−^ Trp53^−/−^* and control mice (Figure S5). Interestingly, we find the expression for many of these p53 target genes did not change between *Aldh2^−/−^ Fancd2^−/−^* and *Aldh2^−/−^ Fancd2^−/−^ Trp53^−/−^* HSPCs, suggesting that the expression of these genes is not solely dependent on p53. Intriguingly, we also find genes that show p53 dependence in certain HSPC subtypes but not others, for example *Sema3f* in LT-HSCs and ST-HSCs, and *Dram1, Ei24, Aifm2,and Slc2a9* in LMPPs. However, the expression differences of these genes failed to achieve statistical significance between *Aldh2^−/−^ Fancd2^−/−^* and *Aldh2^−/−^ Fancd2^−/−^ Trp53^−/−^* due to the low numbers of HSPCs in *Aldh2^−/−^ Fancd2^−/−^* mice. Strikingly, the expression of 16 genes (*Cdkn1a, Eda2r, Phlda3, Bax, Zmat3, Pvt1, Sulf2, Ccng1*, *Bbc3, Perp, Casp1, Aen, Tnfrsf10b, Ctsd, Ier5*, and *Pml*) showed p53-dependent expression in multiple HSPC subtypes, as observed in the expression differences of these genes between *Aldh2^−/−^ Fancd2^−/−^* and *Aldh2^−/−^Fancd2^−/−^ Trp53^−/−^* HSPCs (Figure 5A). We incorporated the expression of these 16 p53 target genes to devise the Haematopoietic (Haem) p53Score in order define a core hematopoiesis-specific p53 signature (Figure 5B). The Haem p53Score is able to quantify p53 activation in HSPCs from both *Aldh2^−/−^ Fancd2^−/−^* and *Aldh2^−/−^ Adh5^−/−^* mice (Figures 5C, 5D, S5B, and S5C). An increased Haem p53Score is also observed in HSPCs from singly deficient in *Aldh2* or *Fancd2* and decrease in the score of HSPCs from *Trp53^−/−^* compared with WT HSPCs, suggesting that the Haem p53Score is sufficiently sensitive to detect basal p53 activity in WT HSPCs. Our Haem p53Score is also able detect increased p53 activity in multiple hematopoietic progenitor populations from human FA patients based on a published dataset (Figure S5E).^52^ Interestingly, the human FA progenitor cells exhibited *MYC* downregulation in p53-activated progenitor cells,^52^ which we also observe in *Aldh2^−/−^ Fancd2^−/−^* HSPCs (Figure S5D). Overall, we show the Haem p53Score can detect p53 activity in both murine and human hema-topoiesis under both DNA damage stress and basal conditions.

### The Haem p53Score identifies human AMLs with adverse survival

Having shown that the p53Score can detect p53 activity in human hematopoietic cells, we now wanted to apply the Haem p53Score to detect p53 activation in human leukemias. We first applied our scoring system to a published scRNA-seq cohort of human acute myeloid leukemia (AML) patients^65^ (Figure 5E). We observe some AML cases (AML556 and AML210A) to exhibit significantly increased Haem p53Score compared with the non-malignant cells from the same patient. In particular, one case (AML916) that harbored an inactivating p53 variant C238Y did not exhibit increased Haem p53Score. To further explore the clinical significance of p53 activity in AML, we interrogated The Cancer Genomic Atlas AML database (TCGA-LAML) that contains 151 AMLs with RNA expression data. The Haem p53Score is on average higher in AMLs with WT (n = 138) compared with mutated (n = 13) TP53 (Figure 5F). Within the AMLs with WT TP53, we find a proportion of AMLs with significantly higher Haem p53Score, implying strong activation of the p53 pathway. Strikingly, we find that the p53-WT AMLs with the highest Haem p53Scores (top 25%) exhibit significantly worse survival compared with the remaining AMLs (hazard ratio 2.34, p = 0.0006) (Figure 5G). Conversely, AML with the lowest Haem p53Scores (bottom 25%) do not appear to confer any additional survival advantage (Figure S6B). AMLs with high Haem p53Score are associated with increased patient age, and prior treatment where chemotherapy was administered to the patient before the clinical sample was harvested for TCGA database (Figure S6C; Table S4). Cox multivariate analysis shows that high Haem p53Score remains an independent risk factor for increased mortality in AML after accounting for the effect of age and prior treatment (Figures 5H and S6E). A subset of AMLs has been shown to downregulate the expression of ALDH2,^66^ but we did not observe an association between ALDH2 expression and p53Score (Figure S6D). In addition, we find that AMLs with high Haem p53Score do not correlate with the European LeukemiaNet (ELN) AML risk classification by genetic driver mutations^67^ (Table S4). However, analysis of the somatic mutations in AML with high Haem p53Score does reveal a significant under-representation of mutations in RUNX1/CEBPA transcription factor complex, as well as mutations and translocations that act through inhibition of differentiation (RUNX1-RUNX1T1, PML-RARA, MLL rearrangements) (Figure 5I; Table S5). In summary, the Haem p53Score identifies a subpopulation of AMLs that endogenously activate their p53 response and is associated with adverse survival. This could have clinical utility in the risk and prognostic stratification of AML.

### Increased p53 activity in aged and myeloid-primed HSPCs

**Figure 6:**
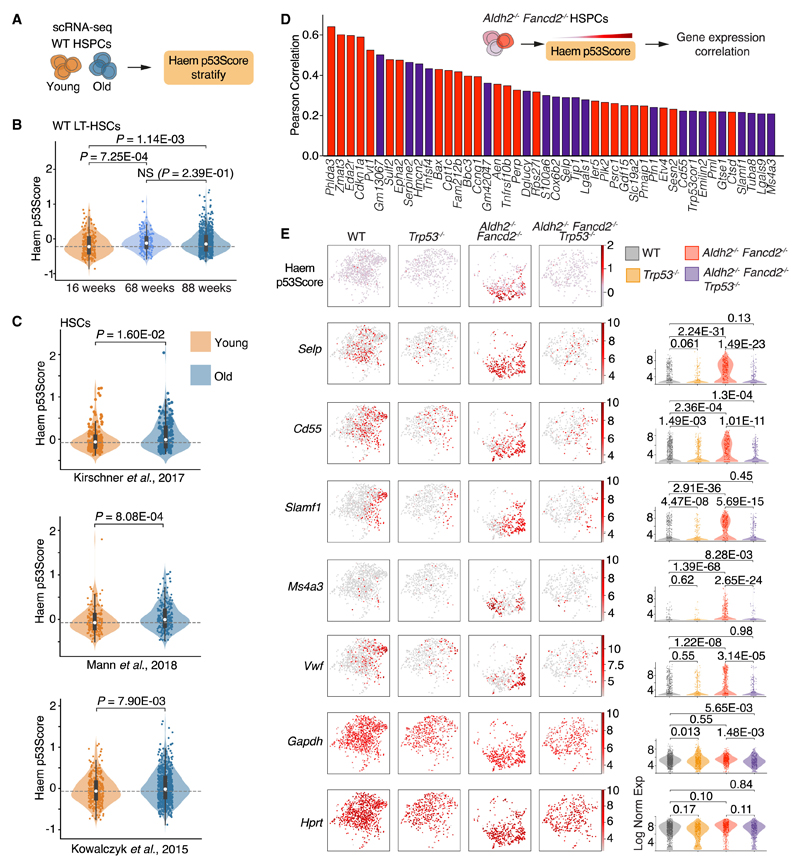
p53 activation in HSPCs correlates with myeloid priming (A and B) Comparison of Haem p53Score in scRNA-seq-derived transcriptomes of LT-HSCs from 16-, 68-week-old WT mice, and an 88-week-old *Fancd2^+–/–^* mouse (n = 234, 187, and 758 left to right), see also Figure S7. (C) Haem p53Score in young and old HSC from three published scRNA-seq datasets.^58,68,69^ (D) Rank of genes by Pearson correlation between gene expression and Haem p53Score in *Aldh2^−/−^ Fancd2^−/−^* LKS cells. Red highlights known p53 gene targets, purple highlights genes not known to be direct p53 targets. See Table S6 for list of genes with Pearson coefficient >1. (E) UMAP and violin plots of LKS cells from WT, *Trp53^−/−^, Aldh2^−/−^ Fancd2^−/−^*, and *Aldh2^−/−^ Fancd2^−/−^ Trp53^−/−^* mice, showing the distribution and scale of Haem p53Score, and respective gene expression that correlate with Haem p53Score. *Gapdh* and *Hprt* are included to show expression of housekeeping genes.

The p53 response is a significant driver of aging in HSPCs, this prompted us to inquire whether older HSPCs exhibited increased p53 activity. We therefore applied the Haem p53Score to transcriptomes of HSPCs from young and old WT mice that reflect physiological aging. Compared with HSPCs from a 16-week-old mouse, HSPCs from 68-week and 88-week-old mice indeed show an elevation of the Haem p53Score (Figures 6A, 6B, and S7). In support of our findings, we also find consistently increased Haem p53Score in HSCs from old mice interrogated from three published studies^58,68,69^ (Figure 6C). Therefore, increased p53 activity is associated with physiologically aged HSCs in old WT mice, as well as prematurely aged HSCs in young *Aldh2^−/−^ Fancd2^−/−^* and *Aldh2^−/−^ Adh5^−/−^* mice. To gain insights into how p53 activation in HSPCs might result in aged and myeloid-biased hematopoiesis, we characterized the genes whose expression correlated with the Haem p53Score of HSPCs from *Aldh2^−/−^ Fancd2^−/−^* mice (Figure 6D; Table S6). As expected, the genes whose expression exhibited the highest correlation with the Haem p53Score were *Trp53* target genes. We also observe a correlation with the top aging marker gene *Selp*. In addition, a group of genes (*Cd55*, *Slamf1*, *Ms4a3*, and *Vwf*) associated with myeloid/megakaryocyte primed HSCs were also correlated with p53 activation (Figure 6E). *Cd55* and *Slamf1* expressing HSPCs have previously been reported to be myeloid and erythroid biased, and in particular Slamf1^high^ HSCs progressively increase with age.^14,70^ In particular, *Cd55* and *Slamf1* expression was downregulated in HSPCs from *Trp53^−/−^* compared with WT mice, indicating that the p53-dependent expression of these genes also operates in homeostatic hematopoiesis. *Ms4a3* expression has been associated with monocytic lineage differentiation,^71^ and enforced expression of *Ms4a3* in chronic myeloid leukemia cells has been shown to induce myeloid differentiation.^72^ Vwf is a marker of the megakaryocyte lineage, and its expression in LT-HSCs delineates a subpopulation of HSCs that are primed toward megakaryocyte and myeloid differentiation.^73,74^ A subset of *Aldh2^−/−^ Fancd2^−/−^* HSPCs also exhibit increased *Vwf* expression, which is lost upon deletion of *Trp53.* Overall, increased p53 activity in *Aldh2^−/−^ Fancd2^−/−^* HSPCs correlates with increased expression of genes associated with aging and myeloid priming.

### Vwf+ LT-HSCs are enriched in *Aldh2^−/−^ Fancd2^−/−^* mice

**Figure 7:**
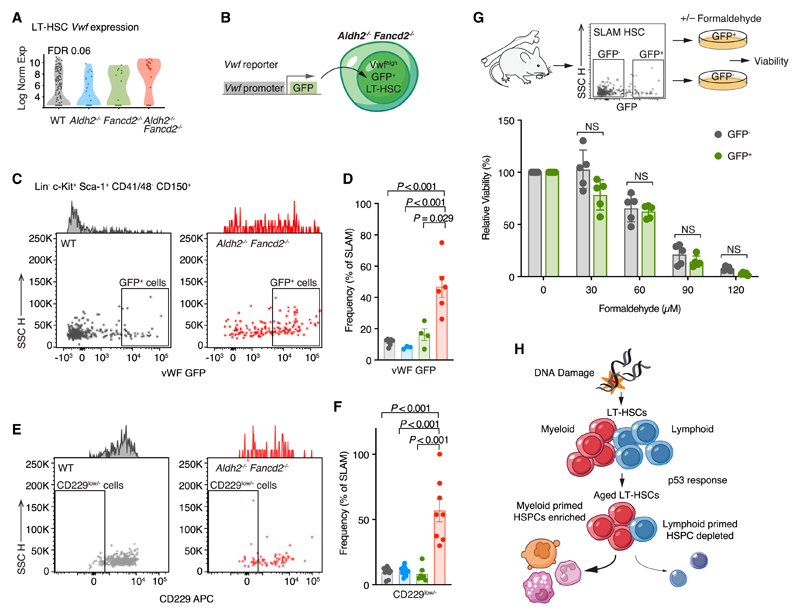
*Aldh2*^−/−^ *Fancd2^−/−^* mice harbor increased Vwf+ LT-HSCs (A) *Vwf* expression in LT-HSC subset of LKS cells analyzed by scRNA-seq. (B) The Vwf-GFP reporter transgene fluorescently labels LT-HSCs that express *Vwf* and was introduced into *Aldh2^−/−^ Fancd2^−/−^* mice and respective controls. (Cand D) GFP^+^SLAM HSCs from WT and *Aldh2^−/−^ Fancd2^−/−^* mice shown by representative flow cytometry plots (C), and bar plot (mean ± SEM; n = 7, 3, 4, and 6, left to right) (D). (E and F) CD229^low/–^ SLAM HSCs from WT and *Aldh2^−/−^ Fancd2^−/−^* mice shown by representative flow cytometry plots (E), and bar plot (mean ± SEM; n = 10, 13, 7, and 8, left to right) (F). (G) *Ex vivo* cultures of Vwf+ and Vwf– SLAM HSCs to assess formaldehyde sensitivity, defined by the number of surviving cells in formaldehyde-supplemented media as a proportion of total cells in untreated media. Each point represents a single mouse with isolated HSCs cultured in technical triplicates. (H) Endogenous DNA damage triggers p53-dependent response in HSCs leading to aging and myeloid bias.

The p53-dependent upregulation of genes associated with myeloid priming in *Aldh2^−/−^ Fancd2^−/−^* HSPCs offers a compelling molecular mechanism for the myeloid-biased hematopoiesis in *Aldh2^−/−^ Fancd2^−/−^* mice. Indeed, we observed increased expression of *Vwf* in *Aldh2^−/−^ Fancd2^−/−^* LT-HSCs (Figure 7A), prompting us to directly quantify the frequency of Vwf+ LT-HSCs in *Aldh2^−/−^ Fancd2^−/−^* mice. To this end, we exploited the Vwf-eGFP transgenic murine reporter that permits identification of Vwf+ LT-HSCs by their GFP fluorescence.^73^ We introduced this reporter into *Aldh2^−/−^ Fancd2^−/−^* and respective WT and single knockout control mice (Figure 7B) to find that the proportion of GFP^+^ SLAM HSCs was significantly elevated in *Aldh2^−/−^ Fancd2^−/−^* HSCs (Figures 7C and 7D). We also find increased the proportion of CD229^low–^ SLAM HSCs in *Aldh2^−/−^Fancd2^−/−^* mice (Figures 7E and 7F), another marker of Vwf+ LT-HSCs.^75^ Taken together, this suggests that elevated endogenous DNA damage in LT-HSCs results in enrichment of Vwf+ LT-HSCs. How does DNA damage shift the LT-HSC compartment to favor Vwf+ and potentially other myeloid-primed HSCs? One possibility could be different genotoxic sensitivity in Vwf+ LT-HSCs compared with Vwf– LT-HSCs. To test this hypothesis, we flow cytometrically sorted equal numbers of Vwf-eGFP^+^ and Vwf-eGFP— SLAM HSCs from WT mice into media containing polyvinyl alcohol (PVA), thrombopoietin (TPO), and stem cell factor (SCF) to maintain LT-HSC viability *ex vivo*.^76^ These cells were then exposed to formaldehyde for 24 h, after which we visually counted the number of cells in culture as a ratio of formaldehyde treated versus untreated cells to determine the relative viability. We found Vwf-eGFP + and Vwf-eGFP– HSCs to be equally sensitive to formaldehyde (Figure 7G). In summary, these results suggest that DNA-damage-driven p53 activation stimulates the accumulation of a subset of myeloid-primed HSCs. This shift in HSC populations may to some extent explain the myeloid-biased output observed in our premature aging driven model.

## Discussion

We have shown that chronic genotoxic aldehyde stress induces fundamental transcriptional, epigenetic, and functional changes in HSCs that recapitulate physiological aging. An important aspect of our study is the use of a physiological model of endogenous DNA damage to alter HSC biology. Furthermore, the use of transcriptional profiling by scRNA-seq enables us to better appreciate the *in situ* effects and consequences of damage, as opposed to changes seen subsequent to transplantation–the cornerstone of functional HSC studies. Our genetic studies implicate a central role for an inducible p53 DDR in driving these changes. Thus, we define an endogenous driver and a mechanistic basis for aging in blood stem cells. We propose a model whereby endogenous aldehydes cause DNA damage in HSCs to trigger p53-dependent aging, leading to enrichment of HSCs primed toward myeloid and megakaryocyte lineages, and depletion of lymphoid-primed HSCs (Figure 7H). Furthermore, we have been able to extract and quantify the HSPC-specific p53 transcriptional response by introducing the Haem p53Score, which can detect increased p53 activity in mouse and human HSPCs. In particular, the Haem p53Score defines a subset of AML with high p53 activation that confers a worse survival. This could contribute to the risk stratification and future treatment of AML.

While prior studies have observed features of early aging in mice harboring aberrant p53 hyperactivity,^77–80^ our study highlights how physiological p53 response impacts hematopoiesis subjected to endogenous DNA damage. We have previously shown that the reduction in HSCs in *Aldh2^−/−^ Fancd2^−/−^* mice is due to p53-dependent attrition.^37^ Our findings here suggest that the p53 response in the HSCs of *Aldh2^−/−^ Fancd2^−/−^* mice is also capable of accelerating the aging response. This supports prior observations of a distinctive role for p53 in HSCs that is independent to activating apoptosis.^80,81^ Interestingly, at a single-cell level, we observe heterogeneity in the metrics of aging in the HSC compartment, with a fraction of *Aldh2^−/−^ Fancd2^−/−^* LT-HSC retaining a young transcriptome signature, minimal telomere attrition and a balanced myeloid-lymphoid reconstitution following transplantation. This heterogeneity in aging could reflect variations in the levels of endogenous DNA damage in individual HSCs, and difference in the DDR between cycling and quiescent HSCs.^82,83^

The role of p53 in HSCs during hematopoiesis under basal conditions is not well defined. In contrast to HSCs challenged with acute DNA damage, prior studies have reported that during homeostatic hematopoiesis, p53-proficient HSCs perform equally or better than p53-deficient HSCs.^81,84,85^ Our study now characterizes p53 in hematopoiesis from WT mice under basal conditions, where we find elevated p53 activity in aged HSCs. This provides a mechanistic link to prior observations of increased markers of DNA damage in naturally aged HSCs.^25,86,87^ One plausible model to account for blood aging could be that the basal metabolic activity of HSCs at homeostasis could generate sufficient endogenous DNA damage to induce low-level, constant p53-driven aging. During periods of elevated genotoxicity that could arise from inflammatory stimuli or exposure to chemoradiotherapy, the increased p53 activity triggers both accelerated aging and attrition of HSC population–as observed in the *Aldh2^−/−^ Fancd2^−/−^* mouse model. Interestingly, it has been shown that the “aged” HSC phenotype is potentially reversible upon sequential transplantation into young recipients.^88^ Other sources of physiological stress such as inflammation have been shown to induce a longer-lived age-related decline and myeloid bias in HSCs.^57^ Currently, we are not able to test whether the maintenance of this aged state requires a sustained p53 response–this would be useful to explore in the future.

A poorly understood feature of aged hematopoiesis is the striking shift in HSC output, which results in fewer lymphoid and more myeloid cell types. The causes and mechanisms that drive this fundamental shift are not yet fully understood. For instance, it is known that this biased output can operate at the very early stem cell level through lineage primed HSCs^14,70,73,88,89^ and can be associated with chronic inflammation^53–57^ and clonal hematopoiesis.^90–94^ Different studies have reported that DNA damage can impact HSC differentiation.^61,62,95^ Our results indicate that chronic endogenous DNA damage induces myeloid-biased output in HSCs by activating the p53 response. The outcome of this p53 response results in predominance of Vwf+ myeloid and platelet-biased HSCs. Indeed, our findings now provide a plausible p53-driven mechanism that underpins the enrichment of Vwf+ HSCs that occurs during aging.^74^ How does p53 influence the heterogeneous composition of the HSC compartment? We did not observe difference in sensitivity to acute formaldehyde toxicity between Vwf+ and Vwf– HSCs cultured in *ex vivo* conditions. However, it is possible that Vwf+ HSCs are more resistant to chronic endogenous DNA damage through better repair or an attenuated p53 response, or due to an active reprogramming mechanism triggered by p53 wherein multipotent HSCs become myeloid-biased Vwf+ stem cells. This will be an important question to address in future studies. What could be the purpose of myeloid-biased blood production in response to DNA damage and p53 activation? It may be an adaptive response to ensure that damaged HSCs are preferentially depleted via differentiation to short-lived yet life-essential blood constituents such as platelets, red cells, and neutrophils. This would reduce the production of longer-lived lymphocytes that could harbor cancer-initiating genomic changes.

Comparing the p53-activated and p53-null transcriptomes in *Aldh2^−/−^ Fancd2^−/−^* and *Aldh2^−/−^ Fancd2^−/−^ Trp53^−/−^* hematopoietic cells allowed us to generate the Haem p53Score to better identify and quantify p53 activity in HSPCs. We envisage this will be a useful research tool in the study of p53 in hematopoiesis. For example, TP53 is often mutated in clonal hematopoiesis, yet *TP53* HSC mutants exhibit the slowest growth rate compared with clones driven by other mutations.^10^ Our dissection of the HSPC p53 transcriptional response could be useful to address the nature of p53 activity in TP53 driven clonal hematopoiesis. We detected increased p53 activity in human AML using the Haem p53Score, and intriguingly find AMLs with high p53 activity to confer worse overall survival. Our findings indicate that the Haem p53Score could have clinical utility for risk stratification of AMLs based on p53 activity, which is not accounted for by the current international standard risk classification by genetics. The biology that underlies the association between increased p53 activity and adverse outcome in AML is a focus for future study. One possible mechanism could be that AML with high p53 activity could have inactivated downstream effectors, thus permits leukemic proliferation and treatment resistance despite vigorous p53 signaling. Another possible explanation for the high p53 activation in AMLs could reflect increased endogenous DNA damage, leading to genomic instability from which p53-null leukemic clones evolve and are selected following chemotherapy. It will be interesting to assess whether AMLs with high p53 activation harbor elevated genotoxic aldehydes, or exhibit deficiency in DNA repair. Such knowledge might contribute to new AML therapeutic strategies at targeting DDR and/or DNA repair machinery.^96,97^

Lastly, our work defines a central role for aldehyde-driven chronic endogenous DNA damage as a driver for HSC aging. Many humans through drinking expose themselves to ethanol, which is a source of acetaldehyde, while our body produces a large amount of formaldehyde. Strategies to limit our exposure to these endogenous aldehydes may improve the functionality and healthy aging of our blood system and potentially other regenerative organ systems.

### Limitations of the study

An important difference between our chronic DNA damage model for HSC aging and the normal aging process is the number of HSCs in the bone marrow. While physiological aging results in increased HSC numbers, in our model, there are very few HSCs. However, in both instances the HSCs show biased outputs and impaired regenerative capacity. While our results indicate a p53-dependent enrichment of Vwf+ HSCs, we cannot ascertain whether this arises from preferential survival and expansion of pre-existing Vwf+ HSCs, or Vwf– HSCs differentiating to contribute to the Vwf+ HSC population. An important question in the p53 field remains how p53 activation decides between diverse cell fates, from irreversible cell death to altered differentiation and cell preservation mechanisms. Our study is unable to address this question, but our characterization of the p53 transcription response in HSCs will lay the foundation to better understand p53-driven decision states in future studies.

## Star★Methods

**Table:** KEY RESOURCES TABLE

REAGENT or RESOURCE	SOURCE	IDENTIFIER
Antibodies		
c-Kit::APC-Cy7 (clone 2B8)	Biolegend	RRID: AB_1626278
Sca-1::BV421 (clone D7)	Biolegend	RRID: AB_2563064
CD45::FITC (clone 30-F11)	Biolegend	RRID: AB_312973
EPCR::PE (clone RMEPCR1560)	STEMCELL Technologies	RRID: AB_1118509
CD48::APC (clone HM48-1)	eBioscience	RRID: AB_469408
CD150::PE-Cy7 (clone TC15-12F12.2)	Biolegend	RRID: AB_439797
CD4::FITC (clone H129.19)	BD Pharmingen	RRID: AB_394970
CD3e::FITC (clone 145-2C11)	Beioscience	RRID: AB_464882
Ly-6G/Gr-1::FITC (clone RB6-8C5)	eBioscience	RRID: AB_465314
CD11b/Mac-1::FITC (clone M1/70)	BD PharMingen	RRID: AB_394774
CD45R/B220::FITC (clone RA3-6B2)	BD PharMingen	RRID: AB_394618
Fc∊R1a::FITC (clone MAR-1)	eBioscience	RRID: AB_465309
CD8a::FITC (clone 53-6.7)	BD PharMingen	RRID: AB_394569
CD11c::FITC (clone N418)	eBioscience	RRID: AB_464941
TER-119::FITC (clone Ter119)	BD PharMingen	RRID: AB_396936
CD41::FITC (clone MWReg30)	BD PharMingen	RRID: AB_1626237
c-Kit::PerCP-Cy5.5 (clone 2B8)	eBioscience	RRID: AB_2534338
Sca-1::PE-Cy7 (clone D7)	eBioscience	RRID: AB_469669
CD150::PE (clone TC15-12F12.2)	Biolegend	RRID: AB_313683
CD48::BV421 (clone HM48-1)	Biolegend	RRID: AB_2650894
CD62P (P-Selectin)::Super Bright 600 (Clone Psel.KO2.3)	Thermo Fisher Scientific	RRID: AB_2802430
CD4::PE (clone RM4-5)	eBioscience	RRID: AB_465509
CD3::PE (clone 145-2C11)	eBioscience	RRID: AB_465496
Ly-6G/Gr-1::PE (clone 1A8)	BD PharMingen	RRID: AB_394208
CD11b/Mac-1::PE (clone M1/70)	BD PharMingen	RRID: AB_396680
CD45R/B220::PE (clone RA3-6B2)	Biolegend	RRID: AB_312992
Fc∊R1a::PE (clone MAR-1)	Biolegend	RRID: AB_1626104
CD8a::PE (clone 53-6.7)	BD PharMingen	RRID: AB_394571
CD11c::PE (clone N418)	eBioscience	RRID: AB_465551
TER-119::PE (clone Ter119)	BD PharMingen	RRID: AB_466041
CD41::PE (clone MWReg30)	BD PharMingen	RRID: AB_397004
CD150::APC (clone TC15-12F12.2)	Biolegend	RRID: AB_493460
CD229/Ly-9::Biotin (clone Ly9ab3)	Biolegend	RRID: AB_830724
CD45.1::BV421 (clone A20)	Biolegend	RRID: AB_2562563
CD45.2::APC (clone 104)	Biolegend	RRID: AB_389211
CD45R/B220::PerCP-Cy5.5 (clone RA3-6B2)	Biolegend	RRID: AB_893354
TER-119::PE-Cy7 (clone Ter119)	Biolegend	RRID: AB_2281408
CD4::Biotin (clone RM4-5)	Biolegend	RRID: AB_312710
CD8a::Biotin (clone 53-6.7)	Biolegend	RRID: AB_312742
CD45R/B220::Biotin (clone RA3-6B2)	Biolegend	RRID: AB_312988
CD127::Biotin (clone A7R34)	Biolegend	RRID: AB_1953262
TER-119::Biotin (clone Ter119)	Biolegend	RRID: AB_313704
Ly-6G/Gr-1::Biotin (clone RB6-8C5)	Biolegend	RRID: AB_313368
c-Kit::APC (clone 2B8)	Biolegend	RRID: AB_313221
Sca-1::PE (clone D7)	Biolegend	RRID: AB_313344
Chemicals, peptides, and recombinant proteins		
Streptavidin::APC-Cy7	Biolegend	Cat#405208
Streptavidin::BV605	Biolegend	Cat#405229
Streptavidin::BV510	Biolegend	Cat#405234
Ammonium Chloride Solution for red cell lysis	StemCell Technologies, Inc.	Cat#07800
7-AAD (7-Aminoactinomycin D)	Thermo Fisher Scientific	Cat#A1310
SUPERase-In™ RNase Inhibitor	Thermo Fisher Scientific	Cat#AM2696
Red Blood Cell Lysis Solution (10x)	Militenyi Biotec	Cat#130-094-183
Benzonase	MilliporeSigma	Cat#E1014
RNase A	Qiagen	Cat#19101
Proteinase K	Qiagen	Cat#19131
Anti-APC Microbeads	Militenyi Biotec	Cat#130-090-855
Ham's F-12 Nutrient Mix liquid medium	Gibco	Cat#11765-054
N-2-hydroxyethylpiperazine-N-2-ethane sulfonic acid (HEPES)	Gibco	Cat#15630-080
Insulin-transferrin-selenium-ethanolamine 100 x	Gibco	Cat#51500-056
Recombinant animal-free murine thrombopoietin (TPO)	Peprotech	Cat#AF-315-14
Recombinant animal-free murine stem cell factor (SCF)	Peprotech	Cat#AF-250-03
Polyvinyl alcohol (PVA), 87-90%-hydrolyzed	MilliporeSigma	Cat#P8136
16% Formaldehyde (w/v)	Thermo Fisher Scientific	Cat#28906
Critical commercial assays		
Lineage Depletion Kit	StemCell Technologies, Inc.	Cat#19816A
Nextera XT DNA sample prep, 96rxn	illumina	Cat#FC-131-1096
Nextera XT Index Kit v2 Set A	illumina	Cat#FC-131-2001
Nextera XT Index Kit v2 Set D	illumina	Cat#FC-131-2004
DNeasy Blood & Tissue Kit	Qiagen	Cat#69504
Qubit dsDNA BR (Broad Range) Assay Kit	Thermo Fisher Scientific	Cat#Q32850
Infinium Mouse Methylation Beadchip	illumina	Cat#20041558
Proinflammatory Panel 1 (mouse) Kits	MesoScale Discovery (MSD)	Cat#K15048D-2
Deposited data		
Single-cell transcriptomes of murine HS(P)Cs	This study	GSE209742
Experimental models: Organisms/strains		
Mouse: *Aldh2^tm1a(EUCOMM)Wtsi^*	EUCOMM	RRID:MGI:5467969
Mouse: *Fancd2^tm1Hou^*	Houghtaling et al.^98^	RRID:MGI:2673422
Mouse: *Trp53^tm1Br^*	Donehower et al.^99^	RRID:MGI:1857590
Mouse: Vwf-eGFP reporter	Sanjuan-Pla et al.^73^	N/A
Software and algorithms		
FlowJo, version 10.6.1	BD Biosciences	N/A
Prism, version 9	GraphPad	N/A
SPSS Statistics, version 28	IBM	N/A
Code and algorithms for analysis	This study	https://doi.org/10.5281/zenodo.7965894
STAR: ultrafast universal RNA-seq aligner, version 2.5.1b	Dobin et al.^100^	http://code.google.com/p/rna-star/
Scanpy, version 1.7.1	Wolfetal.^101^	https://github.com/theislab/Scanpy
Telomerecat	Farmery et al.^63^	https://github.com/cancerit/telomerecat
Cytoscape, version 3.9	Cytoscape Consortium	https://cytoscape.org/index.html
Other		
MACsLS column	Militenyi Biotec	Cat#130-042-401

## Resource Availability

### Lead contact

Further information and requests for resources and reagents should be directed to K.J. Patel, ketan.patel@imm.ox.ac.uk.

### Materials availability

This study did not generate new unique reagents.

## Experimental Model And Study Participant Details

### Mice

All mice were kept in specific pathogen free conditions. All mouse breeding and all experiments undertaken in this study were done so with the approval of the Animal Welfare Ethical Review Body and under project license authority granted by the UK Home Office. *Aldh2^-/-^ Fancd2^-/-^*, and *Aldh2^-/-^ Fancd2^-/-^ Trp53^-/-^* mice were generated on a C57/BL/6 x 129S4S6/Sv F1 hybrid background as previously reported.^34,35^ On the C57BL/6 side, embryonic stem cells carrying the Aldh2 allele was obtained from EUCOMM (Aldh2tm1a(EUCOMM)Wtsi; MGI ID: 4431566, EUCOMM6) and injected into blastocysts from C57BL/6J mice. Chimeric males were bred with C57BL/6J females to obtain germline *Aldh2* transmission, and maintained in the C57BL/6Jo1aJax background. The *Fancd2* allele^98^ (Fancd2tm1Hou; MGI ID: 2673422, 129S4/SvJae, a gift from M. Grompe) had been backcrossed from 129S4/SvJae onto the C57BL/6Jo1a background for 10 generations. The *Trp53* allele^99^ (Trp53tm1Brd; MGI ID: 1857590; 129S7/SvEvBrd-Hprt+) had been backcrossed onto C57BL/6J for six generations. Similarly, on the 129S4S6/Sv side, the *Aldh2* allele (Aldh2tm1a(EUCOMM)Wtsi; MGI ID: 4431566, EUCOMM6) had been backcrossed from C57BL/6Jo1aJax onto 129S6/Sv for five generations. The *Trp53* allele had been backcrossed onto 129S6/Sv for six generations. For each C57BL/6 and 129S4S6/Sv background, mice carrying *Aldh2, Fancd2* and *Trp53* alleles were initially intercrossed within each respective background to generate pure C57BL/6 or 129S4S6/Sv *Aldh2^+/-^ Fancd2^+/-^ Trp53^+/-^* male and females, and *Aldh2^-/-^ Fancd2^+/-^ Trp53^+/-^* males. To generate experimental *Aldh2^-/-^ Fancd2^-/-^* and *Aldh2^-/-^Fancd2^-/-^ Trp53^-/-^* mice in the F1 hybrid background, crosses were set up between 129S4S6/Sv females and C57BL/6 males (or C57BL/6 females crossed with 129S4S6/Sv males). *Aldh2^+/-^ Fancd2^+/-^ Trp53^+/-^* female C57BL/6 or 129S4S6/Sv mice were used to generate F1 hybrids to avoid the maternal *Aldh2^-/-^* detriment on *Aldh2^-/-^ Fancd2^-/-^* feti.^102^ *Aldh2^+/-^ Fancd2^+/-^ Trp53^+/-^* or *Aldh2^+/-^ Fancd2^+/-^ Trp53^+/-^* males were used to generate F1 hybrids. The 16-week- and 68-week-old WT mice, and the 88-week-old *Fancd2^+/-^* mouse used in scRNA-seq analysis of young versus old HSPCs were from the C57BL/6 background.

C57BL/6 mice carrying the bacterial artificial chromosome (BAC) transgenic *Vwf-eGFP* reporter^73^ were a gift from A.R. Green and C. Nerlov. *Vwf-eGFP* reporter mice in the C57BL/6 background were backcrossed to the C57BL/6Jo1aJax background for 2 generations and crossed with *Aldh2^+/-^ Fancd2^+/-^* C57BL/6 mice. Progeny from these crosses were interbred to generate *Aldh2+^/–^ Fancd2+^/–^ Vwf-eGFP* homozygous F0 mice in the C57BL/6 background. These animals were crossed with aforementioned *Aldh2+^/–^ Fand2+^/–^* 129S4S6/Sv to generate *Aldh2^-/-^ Fancd2^-/-^ Vwf-eGFP* heterozygous F1 experimental animals. For flow cytometry experiments, 8-12-week-old *Aldh2^-/-^ Fancd2^-/-^ Vwf-eGFP* heterozygous and control mice from C57BL/6J x 129S6/Sv F1, C57BL/6J, and 129S6/Sv backgrounds were used. For single HSC transplantation experiments, as previously described, F1 C57BL/6J×129S6/ Sv CD45.1 recipients were used. Sample size was not predetermined by statistical methods and animals were not randomized. All mice were given an identification number that was used throughout data collection to blind the investigators. Within experiments, mice were age- and strain-matched.

## Method Details

### Single-cell RNA sequencing

The femurs, tibiae, iliac crest, humeri, and vertebrae of 8-12 weeks old mice were crushed, washed with 10 ml of PBS supplemented with 2 % heat-inactivated FBS, and strained through 70 µm nylon meshes (Falcon). Cell suspension was depleted of red blood cells by ammonium chloride lysis (STEMCELL Technologies), and stained with the lineage depletion kit (19816A, STEMCELL Technologies) following the manufacturer's instructions. Lineage-depleted cells were resuspended in 100 μl of PBS supplemented with 2 % FCS containing the following antibodies against: c-Kit (APC-Cy7, clone 2B8,105826, Biolegend), Sca-1 (BV421, clone D7, 108128, Biolegend), CD45 (FITC, clone 30-F11, 103108, Biolegend), EPCR (PE, clone RMEPCR1560, 60038PE, STEMCELL Technologies), CD48 (APC, clone HM48-1, 17-0481-82, eBioscience) and CD150 (PE-Cy7, clone TC15-12F12.2, 115914, Biolegend). Cells were incubated at 4 °C for 30 minutes in the dark, washed, and resuspended in 100 µl of PBS supplemented with 2 % FCS containing streptavidin (BV510, 405234, Biolegend). Cells were further incubated at 4 °C for 15 minutes, washed and resuspended in 500 μl of PBS supplemented with 2 % FCS containing 0.5 ml 7AAD (A1310, Life Technologies). For HSPCs isolated from *Aldh2^-/-^ Fancd2^-/-^, Aldh2^-/-^ Fancd2^-/-^ Trp53^-/-^* mice and associated controls, the single cells (7AAD^–^ CD45+ lineage^–^ c-Kit^+^ Sca-1^+^ population) were sorted using a Becton Dickinson Influx sorter directly into individual wells of a 96 well PCR plate containing lysis buffer (0.2% Triton X-100 (Sigma), RNase inhibitor (SUPERase. In™ RNase Inhibitor, Thermofisher), and nuclease-free water. cDNA scRNA-Seq libraries were prepared using SmartSeq2 protocol.^40^ Sequencing libraries were prepared using the illumina Nextera XT DNA preparation kit and indexed with Nextera XT Index Kit v2 Set A and D, and sequenced on the illumina Hi-Seq 4000. For the 16-week-, 68-week-old WT mice, and the 88-week-old *Fancd2^-/-^* HSPCs, single cells (7AAD^–^ CD45+ lineage^−^ c-Kit^+^ Sca–1^+^ population) were bulk sorted using a Becton Dickinson Influx sorter, and processed using 10x Chromium (10x Genomics, Pleasanton, CA) according to the manufacturer's protocol. The scRNA-seq of *Aldh2^-/-^ Fancd2^-/-^* HSPCs were performed as described previously.^32^

### Single-cell expression analysis

#### Dataset processing

*Aldh2^-/-^ Fancd2^-/-^ dataset.* Sequenced reads were mapped simultaneously to the Mus musculus genome (mm10) and the 92 ERCC sequences using STAR (version 2.5.1b)^100^ with parameters: –outFilterMultimapNmax=1 and -outSMAunmapped=Within. featureCounts was used to count the number of reads mapped to each gene with parameters t=exon and g=gene_id. Data were pre-processed using the in-house pipeline smqpp package in Python and analyzed using Scanpy (version 1.7.1).^101^ Quality control was applied to exclude low-quality cells (using the parameters fGenes:Total > 15%, mit:mappedToGene < 20%, nNuclearReads > 100K-150K depending on the sample distribution, and ercc:mapped < 20%). Cells were normalized using the EdgeR method^103^ and logged. Cell cycle phases were assigned using the score_genes_cell_cycle function based on genes described in ^69^. The normalized log-transformed data was scaled before score calculation. Highly variable genes were selected based on the Brennecke method^104^ using the ERCCs. To have a better overview of the biological information, cell cycle phase effects were removed using the regress_out function. *Aldh2^-/-^ Adh5^-/-^ dataset:* The data was downloaded from GEO accession: GSE157832, normalized to a final total count of 10K for each cell and logged. The cell phase for each cell was estimated using the function score_ genes_cell_cycle in Scanpy. Highly variable genes were selected using the highly_variable_genes function in Scanpy with min_ mean=0.02, max_mean=3, min_disp=0.3 and batch effects were considered. *Human FA and healthy volunteer HSPC dataset:* The data was downloaded from GEO accession: GSE157591 which includes 5 healthy and 6 patients samples. To be consistent with the original study,^52^ CD34+ HSCPs were further selected with CD34 gene expression (normalized to a final total count of 10K) > 0.125. The dataset was normalized to a final total count of 10K for each cell and logged. In total, there were 3312 cells selected. Highly variable genes were selected using the highly_variable_genes function in Scanpy with min_mean=0.02, max_mean=3, min_disp=0.5 and batch effects were considered. Data were then integrated using harmony. *Young and aged murine HSPC datasets:* For the 16-week-, 68-week-old WT mice, and the 88-week-old *Fancd2^+/-^* HSPCs, raw reads were mapped to the Mus musculus genome (mm10) and quantified using the Cell Ranger pipeline (version 6.0.1) with default parameters. Cell-associated barcodes and background-associated barcodes were determined using the EmptyDrops method implemented in the Cell Ranger pipeline, and the background-associated barcodes were excluded. Subsequent data analysis was performed using Scanpy (version 1.8.1). The cells were filtered with min_genes=1500 using the filter_cells function in Scanpy and cells with percentage of mitochondrial reads > 10% were further removed. Multiplets were estimated using the Python package Scrublet and subsequently removed. The dataset was normalized to 10K and logged. Additional young and aged mouse HSC scRNA-seq datasets were extracted from GEO accession: GSE87631,^68^ GEO accession: GSE100426,^58^ and GEO accession: GSE100426.^69^ The cells were filtered with min_genes=800 using the filter_cells function in Scanpy and cells with total counts > 3M or percentage of mitochondrial reads > 10% were further removed. Non-expressed genes across all the cells were removed. The cells were normalized to a total count of 10K per cell and logged.

#### UMAP visualization and HSPC cell-type assignment

*Aldh2^-/-^ Fancd2^-/-^* dataset: The processed data were scaled and top 50 PCA components were calculated. Uniform Manifold Approximation and Projection (UMAP) was used for visualisation using the umap function in Scanpy. In order to acquire the cell type information for each cell, the LKS samples from all genotypes were projected onto 2 different pre-annotated reference landscapes: 1) Nestorowa et al. describing hematopoietic stem and progenitor populations,^51^ and 2) Dahlin et al., containing both LKS and LK populations.^50^ To project the acquired transcriptomes onto the reference landscapes, we used PCA projection. Highly variable genes from the reference landscape were used for the PCA calculation. The 15 nearest neighbor cells from the reference landscape were calculated for each cell using Euclidean distance, and the cell type annotation was assigned as the most frequent cell type out of the 15 matched nearest neighbors. As the dataset described by Nestorowa et al. contains more detailed annotation of hematopoietic stem and earliest progenitor cells whereas the landscape from Dahlin et al. captures a higher cells number and broader snapshot of HSPCs, for the final cell type assignment, the Dahlin landscape was used as the key reference, from which the cells that were annotated as HSC or immature populations were then additionally assigned according to the transcriptomic profiles of cellular states from the Nestorowa landscape. *Aldh2^-/-^ Adh5^-/-^* dataset: Leiden clustering was performed with resolution = 1 and resulted in 16 clusters. Cell annotation was done by projecting the new data onto the PCA space of the Dahlin LK landscape.^50^ Cells annotated as 'HSC' and 'Immature' were selected as HSPCs. To further eliminate outlier cells, HSPCs were restricted within clusters 0, 1 and 5. Effects of the number of counts, the percentage of mitochondrial reads and cell phases were removed using the regress_out function in Scanpy. Leiden clustering was done on HSPCs with resolution = 0.5, which results in 6 clusters and their annotation were defined by the expression profile of key lineage marker genes, including Procr, Mllt3, Mettl7a1, Flt3, Dntt, Mpo, Ctsg and Cdk6. *Human FA and healthy volunteer HSPC dataset:* Leiden clustering was performed with resolution=1.5 and this resulted in 21 clusters. These clusters were annotated based on Velten et al.,^105^ into 12 different cell types. *Young and aged murine HSPC datasets:* For the 16 weeks and 68 weeks old WT mice, and the 88 weeks old *Fancd2+^/–^* HSPCs, the dataset was projected onto the PCA space of the Dahlin LK landscape first. Cells annotated as 'HSC' and 'Immature' were further projected onto the Nestorowa landscape to further specify the LKS subpopulations.

#### Differential gene expression analysis

Differential expression analysis was performed using the edgeR package^103^ and formed the basis for (Kyoto Encyclopedia of Genes and Genomes) KEGG and GO pathway enrichment analysis. When conducting differential expression analysis between two genotypes, to eliminate the confounding factors from differences in cell phase and cell types, these two variables were modelled with the design matrix as ˜Condition(genotype)+phase(cell cycle)+Combined_CT(cell type). To gain a more consistent view of the expression of genes that matched the linear model, log counts per million (logcpm) were extracted and used for downstream plotting. All plotting was performed using either ggplot2 in R or matplotlib in Python. For violin plots, pair-wise adjusted p values were calculated using the two-sided Mann-Whitney-Wilcoxon test with Bonferroni correction.

#### Aging signature score

The aging signature score was calculated using the expression of top 20 (*Selp*, *Mt1*, *Nupr1*, *Plscr2*, *Clec1a*, *Gstm2*, *Enpp5*, *Itgb3*, *Mt2*, *Clca3a1*, *Zg16*, *Sbspon*, *Trpc1*, *Gpr183*, *Klhl4*, *Ptprk*, *Vwf*, *Cd38*, *Neo1*, *Fhl1*) of the 220 Aging Signature genes^46^ using the score_genes function in Scanpy that calculates the average expression of a set of genes subtracted with the average expression of a randomly sampled reference set of genes.

#### Haem p53Score

The Haem p53Score was calculated using the expression values of 16 *Trp53* target genes that exhibit p53-dependent pattern of expression in LKS cells (*Cdkn1a, Eda2r, Phlda3, Bax, Zmat3, Pvt1, Sulf2, Ccng1, Bbc3, Perp, Casp1, Aen, Tnfrsf10b, Ctsd, Ier5, and Pml*) using the score_genes function in Scanpy that calculates the average expression of a set of genes subtracted with the average expression of a randomly sampled reference set of genes. The 694 randomly selected reference genes are found in Table S7. The Haem p53Score was applied to HSPC cells from all genotypes, as well as published datasets: *Aldh2^-/-^ Adh5^-/-^* scRNA-seq data extracted from GEO accession: GSE157832,^32^ human AML scRNA-seq data extracted from GEO accession: GSE116256,^65^ young and aged mouse HSC scRNA-seq extracted from GEO accession: GSE87631,^68^ GEO accession: GSE100426,^58^ and GEO accession: GSE100426.^69^

#### Flow cytometry

For analysis of the HSC compartment, bone marrow cells were harvested from femurs, tibiae, hips, and spine with staining buffer (ice cold PBS 2% + FBS) and strained through 70 µm nylon strainers (Falcon). Red cell lysis was performed for 10 minutes using red cell lysis solution (130-094-183; MACS Militenyi Biotec). Cells were pelleted and resuspended in counting buffer consisting of 3% solution of acetic acid with methylene blue for counting nucleated cells using the Vi-Cell XR cell viability counter (Beckman Coulter). 10 x 10^6^ bone marrow cells were resuspended in 200 ml of PBS supplemented with 2 % FCS containing antibody solution to identify Selp+ or Vwf^+^ SLAM HSCs. The Selp-HSC panel contained FITC-conjugated lineage cocktail with antibodies against CD4 (clone H129.19, BD Pharmingen), CD3e (clone 145-2C11, eBioscience), Ly-6G/Gr-1 (clone RB6-8C5, eBioscience), CD11b/Mac-1 (clone M1/70, BD Pharmingen), CD45R/ B220 (clone RA3-6B2, BD Pharmingen), FcɛR1α (clone MAR-1, eBioscience), CD8a (clone 53-6.7, BD Pharmingen), CD11c (clone N418, eBioscience), TER-119 (clone Ter119, BD Pharmingen), CD41 (FITC, clone MWReg30, BD Pharmigen); c-Kit (PerCP-Cy5.5, clone 2B8, eBioscience), Sca-1 (PE-Cy7, clone D7, eBioscience), CD150 (PE, clone TC15-12F12.2, Biolegend), CD48 (BV421, clone HM48-1, Biolegend), and CD62 P-selectin (Selp) (Superbright600, clone PSEL.KO2.3, invitrogen). For Vwf-eGFP^+^ SLAM HSC staining, we used a PE-conjugated lineage cocktail (CD4 (clone RB6-8C5, eBioscience), CD3e (clone 145-2C11, eBioscience), Ly-6G/Gr-1 (clone 1A8, BD Bioscience), CD11b/Mac-1 (clone M1/70, BD Bioscience), CD45R/B220 (clone RA3-6B2, Biolegend), FcsR1α (clone mar1, Biolegend), CD8a (clone 53-6.7, BD Bioscience), CD11c (clone n418, eBioscience), Ter-119 (clone Ter119, BD Bioscience), CD41 (clone MWReg30, BD Pharmigen)), c-Kit (PerCP-Cy5.5, clone 2B8, eBioscience), Sca-1 (PE-Cy7, clone D7, eBioscience), CD150 (APC, clone TC15-12F12.2, Biolegend), CD48 (BV421, clone HM48-1, Biolegend), CD229 (Biotin, clone Ly9ab3, Biolegend) and Streptavidin (BV605, Biolegend), avoiding the usage of FITC conjugated antibodies to be able to detect GFP fluorescence. After staining, cells were pelleted, washed with 2mL staining buffer, and resuspended in 300-500 μL running buffer (PBS + 2% FBS, Benzonase 0.25 units/mL (Merck, E1014), 5 mM MgSO_4_). Cells were run on the LSRII FACS analyzer (BD Pharmigen) and analyzed with FlowJo (BD Biosciences; Version 10.6.1). LKS cells were defined as lineage^−^ CD41^−^ Sca-1^+^ c-Kit^+^ and HSCs were defined as LKS CD48^−^ CD150^+^.

#### DNA methylation age analysis

Bone marrow cells were flushed from femurs, tibiae, and hips, with ice cold PBS and strained through 70 μm nylon strainers (Falcon). Red cell lysis buffer (130-094-183; MACS Militeny Biotec) was applied for 10 minutes. Cells were pelleted and resuspended in ice cold PBS and counted. 5 × 10^6^ total bone marrow cells were harvested for genomic DNA extraction using Qiagen DNeasy Blood and Tissue kit (69504; Qiagen) as per protocol including treatment with Proteinase K (19131; Qiagen) and RNase A (19101; Qiagen). Eluted DNA was quantified using Qubit dsDNA BR Assay (Q32850; ThermoFisher Scientific) on Qubit4 Fluorometer as per manufacturer's instructions. Minimum of 250 ng DNA was used for DNA methylation profiling on the Infinium Mouse Methylation Beadchip (20041558; Illumina) as per manufacturer's instructions. DNA methylation age was extracted as previously described.^106^ DNA methylation profiling and bioinformatic analysis was performed by Cambridge Genomic Services.

#### Single HSC transplants

Single HSC transplants were performed as described previously, and includes reanalysis of a subset of previously published single HSC transplants derived from WT, *Aldh2^-/-^, Fancd2^-/-^*, and *Aldh2^-/-^Fancd2^-/^* mice.^37^ Chimerism was determined at 16 weeks post-transplant using flow cytometry on tail-vein drawn blood. All recipients with CD45.2^+^ chimerism above 0.005% of total CD45^+^ blood cells (which includes cells derived from the CD45.1^+^ recipient or carrier cells. were included in the final analysis. TER-119 was used to exclude red blood cell debris and chimerism was calculated for each of the WBC lineages (B220^+^ B cells, CD4^+^ CD8^+^ T cells and Gr-1^+^ Mac-1^+^ myeloid cells) as the proportion of total CD45.2^+^ cells.

#### Serum analysis

For serum analysis, whole blood was harvested from mice by cardiac puncture. Serum was separated from whole blood by centrifugation in Microvette 200 conical tubes (MCV200- SER) at 10,000 rpm for 5 minutes. Serum was frozen immediately and stored at -80 °C for analysis. The Proinflammatory Panel 1 (mouse) (K15048D-2; MesoScale Discovery (MSD)) was used to profile proinflammatory cytokines. Measurements were performed by the Cambridge Biochemical Assay Laboratory (CBAL).

#### Telomere estimation

Telomere length was estimated from the ratio of telomeric to sub-telomeric reads by applying the algorithm Telomerecat^11,63^ on the whole genome sequences of peripheral blood mononuclear cells derived from singly transplanted LT-HSCs as previously described.^37^

#### TCGA-AML dataset analysis

The TCGA-LAML dataset^107^ was accessed from National Institute of Health (NIH) Genomic Data Commons (GDC),^108^ of which 151 cases had RNA sequencing data available for analysis using the Haem p53Score. Clinical information including survival and karyotype was obtained from the cBio Cancer Genomics Portal (cBioPortal).^109^ AML somatic driver mutations including TP53 mutation was retrieved from previous analysis of TCGA-LAML RNA sequencing using RNAmut.^110^ The driver mutations and karyotype information was used to risk stratify the TCGA-AML cases following using the 2022 European Leukemia Net risk stratification by genetics at initial diagnosis.^67^

#### Vwf HSC *ex vivo* culture and formaldehyde treatment

The protocol was adapted from a previous study.^76^ In brief, the spine, femurs, tibiae and iliac crests were dissected, cleaned of muscle, ground in ice cold PBS to release bone marrow cells. The harvested cells were strained through 70 µm nylon strainers (Falcon) and stained with 0.2 µl of anti-c-Kit (APC, clone 2B8, Invitrogen) per 10 million cells for 30 minutes at 4 °C, then washed in PBS and incubated for 15 minutes with 0.2 µl anti-APC microbeads (Miltenyi Biotec, 130-090-855) per 10 million cells. Cells were then washed and strained through 70 µm nylon strainers (Falcon) into a pre-PBS-rinsed MACs LS column (Miltenyi Biotec, 130-042-401) on a magnetic stand. The column was washed three times with 3 ml PBS before elution in 5 ml PBS. c-KIT enriched bone marrow cells was stained with 3 uL cocktail of biotinylated lineage antibodies (Biolegend) diluted in PBS containing antibodies against CD4 (clone RM4-5, 1:28), CD8 (clone 53-6.7, 1:28), CD45R/B220 (clone RA3-6B2, 1:14), CD127 (clone A7R34, 1:14), TER-119 (clone TER-119, 1:7) and Ly6G/ Gr-1 (clone RB6-8C5, 1:7) per 10 million cells. After 30 minutes, the cells were washed in PBS and incubated for 90 minutes with antibody cocktail against c-Kit (APC, clone 2B8, Invitrogen), Sca-1 (PE, clone D7, Biolegend), CD150 (PE-Cy7, clone TC15-12F12.2, Biolegend), CD48 (BV421, clone HM48-1, Biolegend) and Streptavidin (APC-Cy7, Biolegend). Finally, the cells were washed, resuspended in PBS containing 1 mg/ml propidium iodide and sorted using a FACS AriaII (BD). GFP^+^ or GFP^−^ LKS CD48^−^ CD150^+^ cells were sorted as previously described,^73^ 50 cells per well, into round-bottom 96-well plates containing 200 mL of F12 media (11765-054; Gibco) supplemented with 1 mg/ml PVA, 100 ng/ml TPO, 10 ng/ml SCF, 0.01 M N-2-hydroxyethylpiperazine-N-2-ethane sulfonic acid (HEPES) and insulin–transferrin–selenium–ethanolamine. Following 24 hours culture at 37 °C, 5 % oxygen, 5% carbon dioxide, the cells were imaged using an AMG Evos microscope and manually counted. Formaldehyde was diluted in F-12 media to five times working concentration and added atop existing media to one times final concentration. After 48 hours of treatment, the cells were imaged, counted, and the percentage survival was calculated by the number of formaldehyde-treated cells as proportion of untreated cells.

### Quantification And Statistical Analysis

The number of independent biological samples is shown as n. Data is represented as mean + SEM. Unless otherwise stated in the figure legend, a two-sided nonparametric Mann-Whitney test was used to determine statistical significance. Analysis was performed using GraphPad Prism Version 9. Chi-square tests and multivariate Cox regression analysis on the TCGA-AML dataset was performed using IBM SPSS Statistics Version 28. Kaplan Meier survival analysis and Hazard Ratio by logrank method was performed using GraphPad Prism Version 9.

## Supplementary Material

Supplementary Material

## Data Availability

The data corresponding to the single-cell RNA-Seq is deposited in the Gene Expression Omnibus database (GEO accession: GSE209742) and are publicly available as of the date of publication. All original code has been deposited at Zenodo and is publicly available as of the date of publication. DOI is listed in the key resources table. Any additional information required to reanalyze the data reported in this paper is available from the lead contact upon request.

## In brief

Wang et al. show that DNA damage caused by genotoxic aldehydes in HSCs results in accelerated aging and differentiation bias to myeloid cells. This HSC aging is driven by the p53 pathway and indicates that aging in the blood system can arise as a consequence of the DNA-damage response.

## Highlights

- Genotoxic aldehydes in HSCs cause accelerated aging and myeloid bias
- Aged HSCs show increased activation of p53 response
- p53 activation is associated with enrichment of myeloid-biased HSCs
- Deletion of p53 rescues HSCs from aging and myeloid bias
