# Supplementary material for: Genotoxic aldehyde stress prematurely ages hematopoietic stem cells in a p53-driven manner
Source: Mol Cell. Author manuscript; Available in PMC 2023 Aug 4. (PMC7614878; doi:10.1016/j.molcel.2023.05.035)
Supplement: Supplementary Material [file EMS182024-supplement-Supplementary_Material.pdf]

**Molecular Cell, Volume *83***

## Supplemental information

# Genotoxic aldehyde stress prematurely ages hematopoietic stem cells in a p53-driven manner

**Meng Wang, Laura T.L. Brandt, Xiaonan Wang, Holly Russell, Emily Mitchell, Ashley N. Kamimae-Lanning, Jill M. Brown, Felix A. Dingler, Juan I. Garaycoechea, Tomoya Isobe, Sarah J. Kinston, Muxin Gu, George S. Vassiliou, Nicola K. Wilson, Berthold Göttgens, and Ketan J. Patel**

# SUPPLEMENTAL TEXT AND FIGURES

# Genotoxic aldehyde stress prematurely ages hematopoietic stem cells in a p53-driven manner

**Authors:** Meng Wang[1,2,3,8,*], Laura T. L. Brandt[3,8], Xiaonan Wang[2,4,8], Holly Russell[7], Emily Mitchell[2,5], Ashley N. Kamimae-Lanning[7], Jill M. Brown[7], Felix A. Dingler[7], Juan I. Garaycoechea[6], Tomoya Isobe[2], Sarah J. Kinston[2], Muxin Gu[2], George S. Vassiliou[2], Nicola K. Wilson[2], Berthold Göttgens[2], Ketan J. Patel[7,9,*]

**Affiliations:**

[1] Division of Nutritional Sciences, Cornell University, Ithaca, USA

[2] Wellcome-MRC Cambridge Stem Cell Institute, Jeffrey Cheah Biomedical Centre, University of Cambridge, Cambridge, UK

[3] MRC Laboratory of Molecular Biology, Francis Crick Avenue, Cambridge, UK

[4.] School of Public Health, Shanghai Jiaotong University School of Medicine, Shanghai, China

[5] Wellcome Sanger Institute, Hinxton, UK

[6] Hubrecht Institute-KNAW (Royal Netherlands Academy of Arts and Sciences) and University Medical Center, Utrecht, Netherlands

[7] MRC Weatherall Institute of Molecular Medicine, University of Oxford, John Radcliffe Hospital, Oxford, UK

[8] These authors contributed equally

[9] Lead contact

*Correspondence:

mengwang@cornell.edu (M.W.), ketan.patel@imm.ox.ac.uk (K.J.P.)

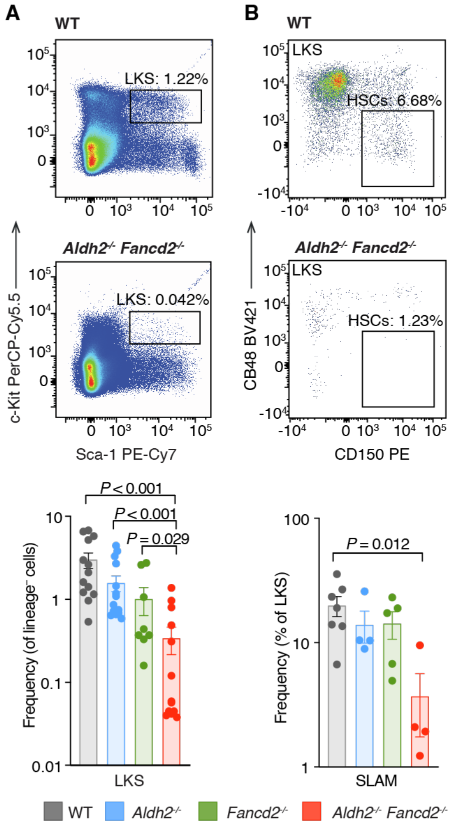


**Figure S1, relating to Figure 1. Loss of HSPCs in *Aldh2*$^{-/-}$ *Fancd2*$^{-/-}$ mice**

Representative flow cytometry plots and quantification from WT and *Aldh2*$^{-/-}$ *Fancd2*$^{-/-}$ mice showing A. LKS (mean ± SEM, n = 13, 15, 8, 13, left to right) and B. SLAM HSCs (mean ± SEM; n = 7, 4, 5, 4, left to right).

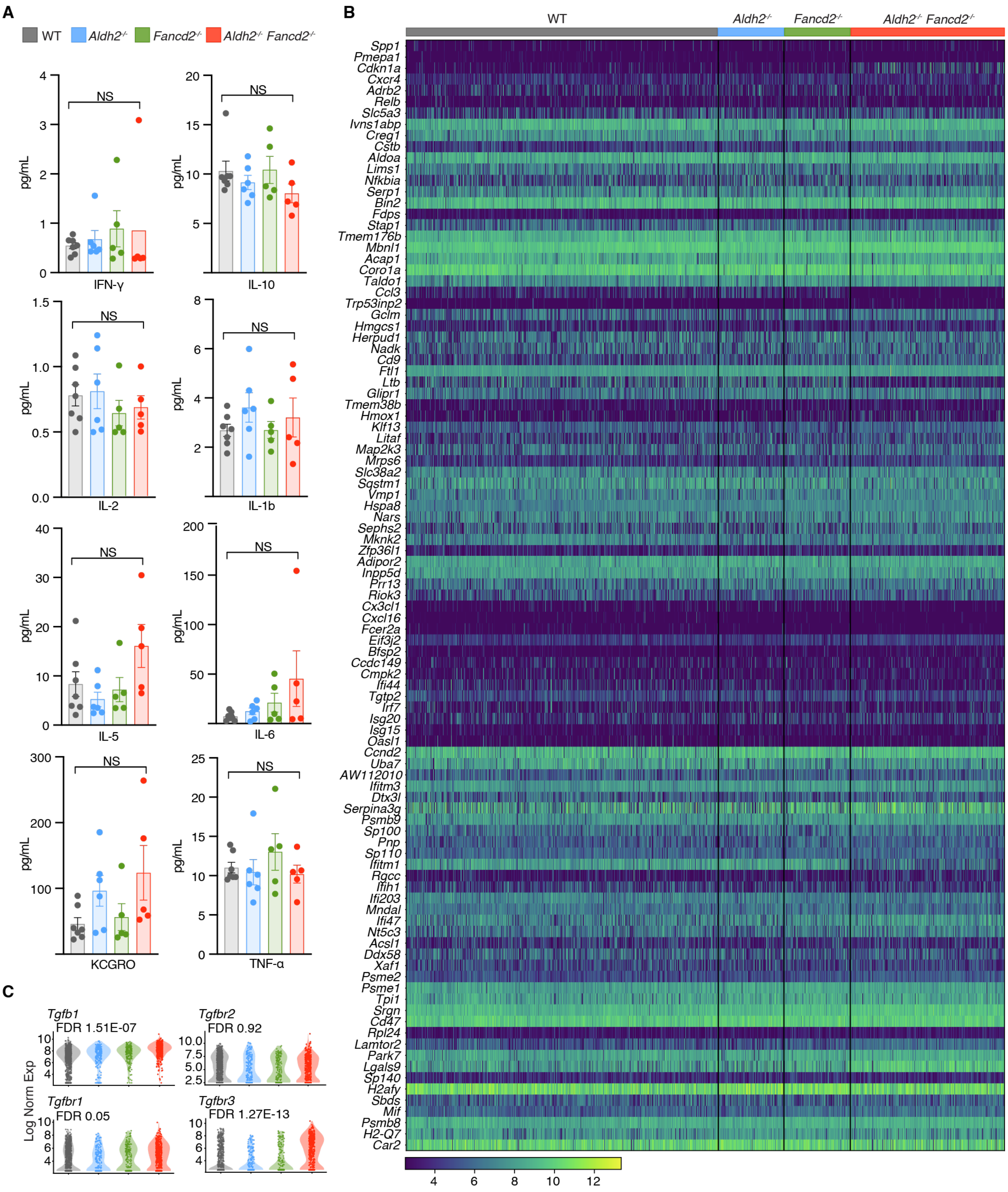


**Figure S2, relating to Figure 3. Inflammation transcription signature and serum cytokine profile**

A. Serum cytokine levels from *Aldh2*$^{-/-}$ *Fancd2*$^{-/-}$ and control mice (mean ± SEM, n = 7, 6, 5, 5, left to right). B. Expression heatmap of gene-set previously shown to be differentially expressed in HSPCs upon inflammatory challenge with

lipopolysaccharide exposure [58]. C. Expression of *Tgfb1*, *Tgfbr1*, *Tgfbr2*, *Tgfbr3* in LKS cells of WT, *Aldh2*$^{-/-}$, *Fancd2*$^{-/-}$ *and Aldh2*$^{-/-}$ *Fancd2*$^{-/-}$ mice. FDR represents comparison between WT and *Aldh2*$^{-/-}$ *Fancd2*$^{-/-}$.

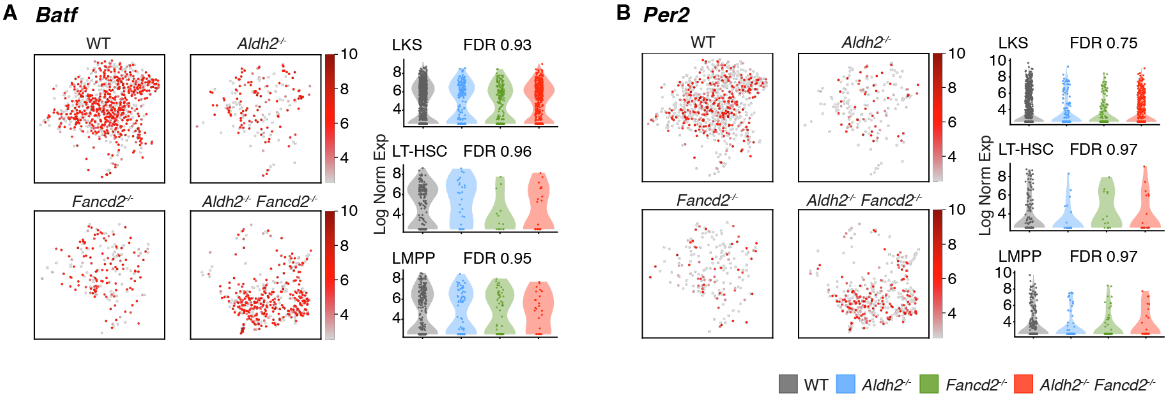


**Figure S3, relating to Figure 3. Absence of *Batf* and *Per2* upregulation in two tier deficient HSPCs**

A. *Batf*, B. *Per2* expression shown on UMAP of LKS cells and violin plots of LKS cells, LT-HSCs and LMPPs from *Aldh2*$^{-/-}$ *Fancd2*$^{-/-}$ and control mice. FDR represents comparison between WT and *Aldh2*$^{-/-}$ *Fancd2*$^{-/-}$.

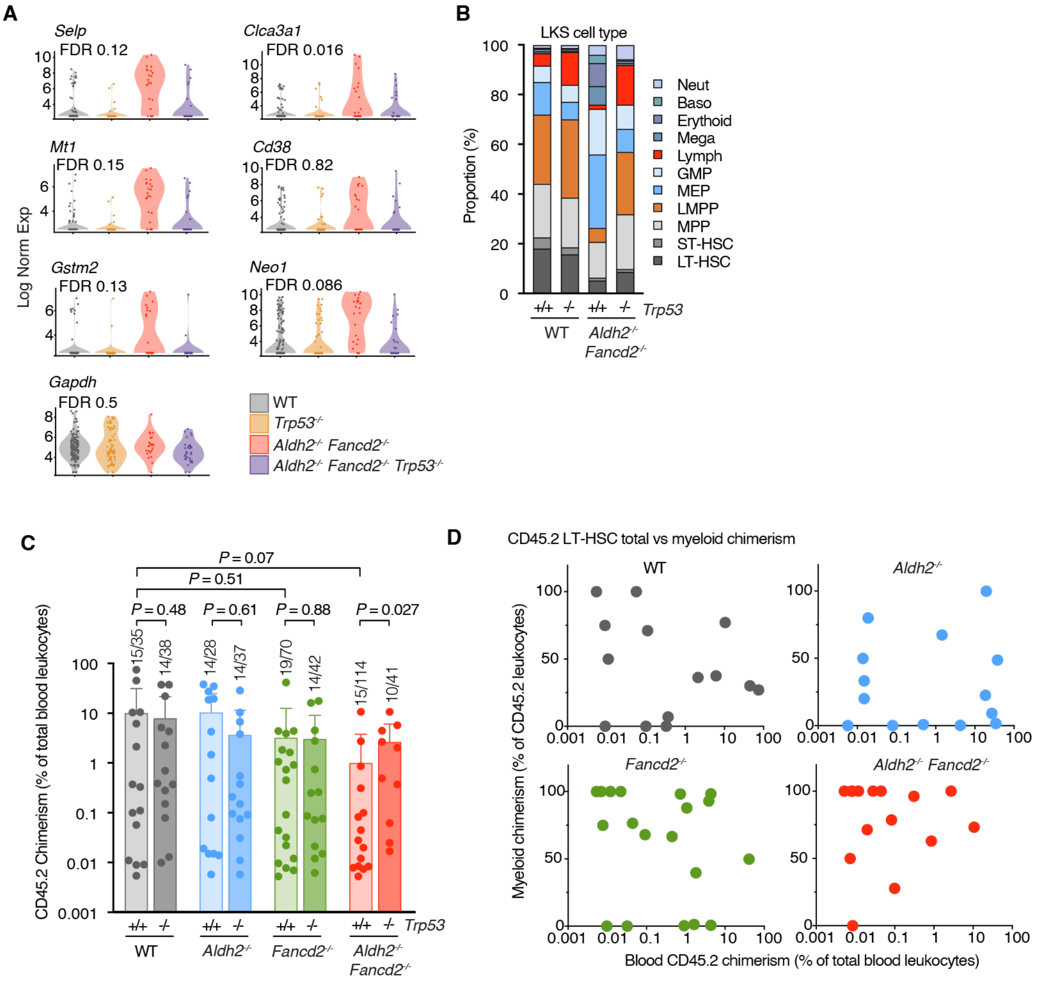


**Figure S4, relating to Figure 4. Aging signature gene expression and myeloid bias depend on p53**

A. Expression of aging-associated genes in LT-HSCs (n = 162, 71, 23, 33, left to right, FDR represents comparison between *Aldh2$^{-/-}$ Fancd2$^{-/-}$* and *Aldh2$^{-/-}$ Fancd2$^{-/-}$ Trp53$^{-/-}$*). B. LKS cell types identified based on transcriptome identity as a proportion of total LKS cells. C. Chimerism of singly transplanted CD45.2$^{+}$ HSCs, quantified by the number of CD45.2$^{+}$ peripheral blood leukocytes as a proportion to total (CD45.1$^{+}$ + CD45.2$^{+}$) peripheral blood leukocytes (mean ± SD; n = 15, 14, 14, 14, 19, 14, 15, 10, left to right). D. Correlation of the engraftment level of each singly transplanted HSC (CD45.2$^{+}$ chimerism), with its resultant myeloid progeny output (myeloid chimerism).

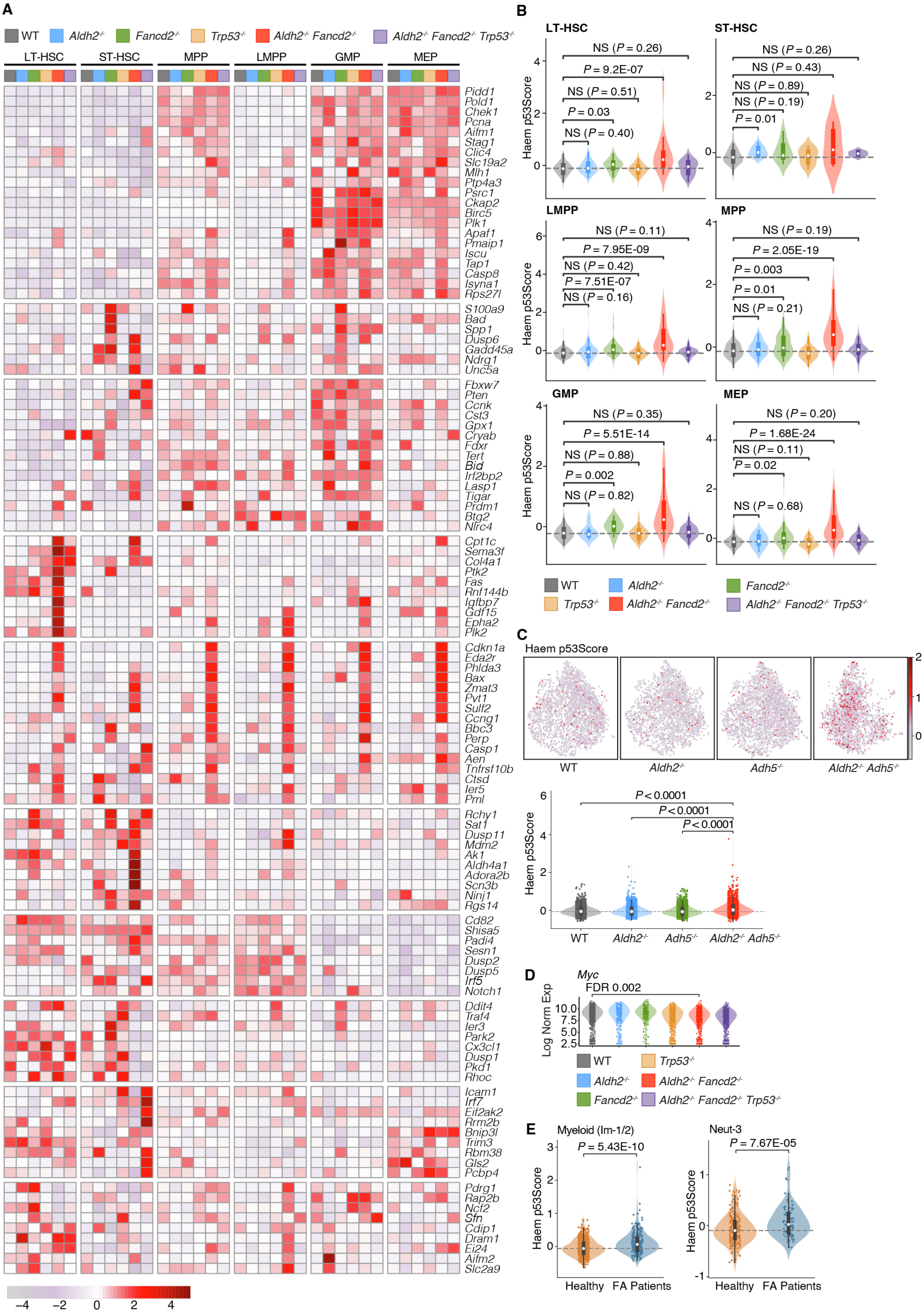


**Figure S5, relating to Figure 5. Expression of p53 target genes in HSPCs**

A. Expression heatmap of 113 validated p53 target genes [64]. B. Distribution of Haem p53Score in LT-HSC, ST-HSC, MPP, LMPP, GMP and MEP populations. C. Haem p53Score of LKS cells from *Aldh2*$^{-/-}$*Adh5*$^{-/-}$ and control mice shown on UMAP and violin plot. D. Expression of *Myc* in LKS cells. E. Haem p53Score of myeloid progenitors lm-1/2 and neut-3 in human healthy volunteers and FA patients.

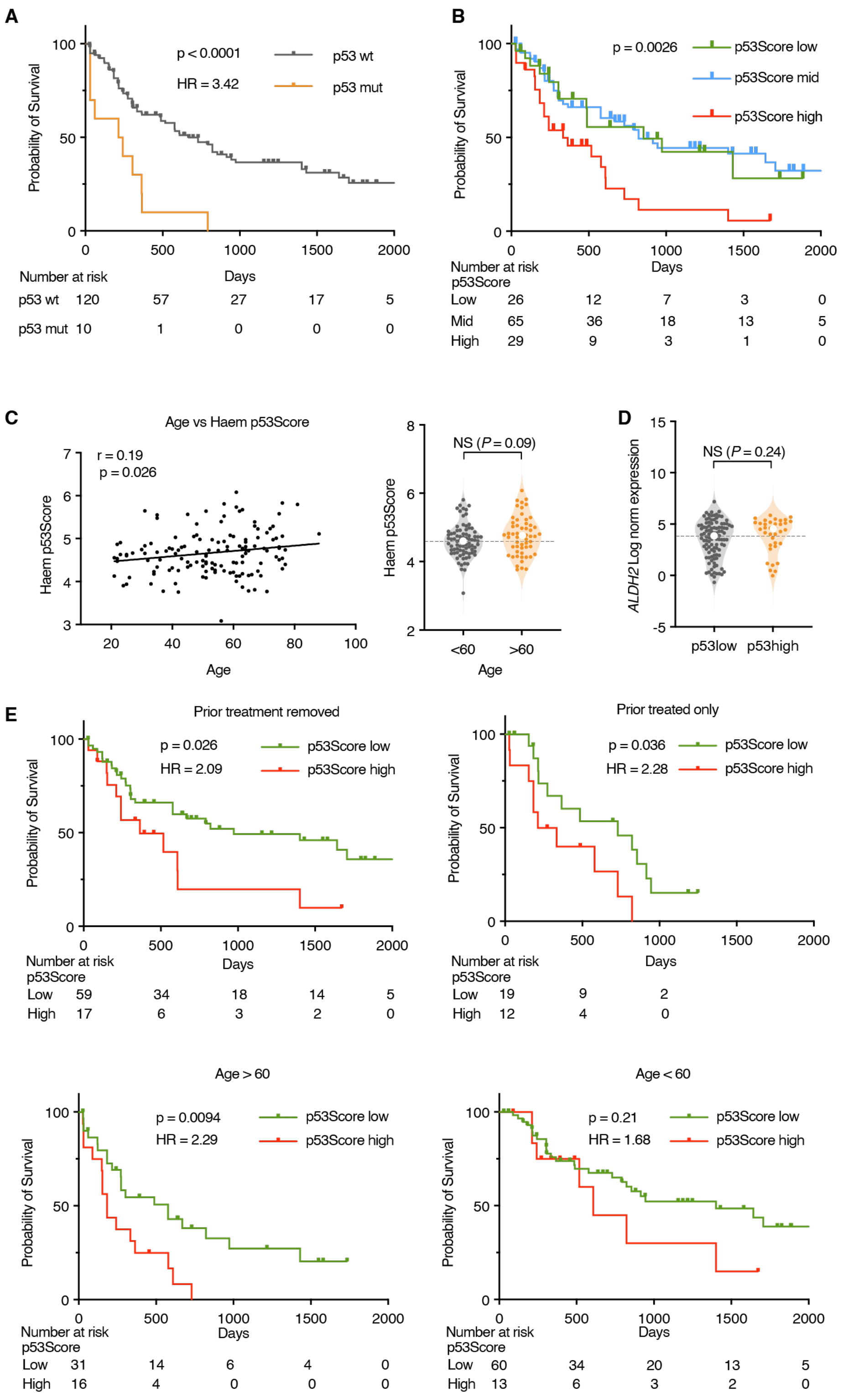


**Figure S6, relating to Figure 5. Haem p53Score in AML correlates with worse outcome**

A. KM survival analysis of TCGA AML cases with wildtype (n = 138) or mutated (n = 13) TP53. HR = Hazard ratio of increased risk of death in *TP53* mutated AMLs. B. KM survival analysis of *TP53* wildtype TGCA AML cases (n = 138) stratified by high (top 25%), mid (middle 50%) or low (bottom 25%) of all Haem p53Score. C. Analysis of AML Haem p53Score correlation with patient age shown by Scatter plot (r = Pearson correlation) and stratification of patients aged under and over 60. D. Expression of *ALDH2* in AML with high and low Haem p53Score. E. Prior treatment and age subgroup survival analysis of *TP53* wildtype AMLs stratified by high (top 25%) and low p53Score (bottom 75%).

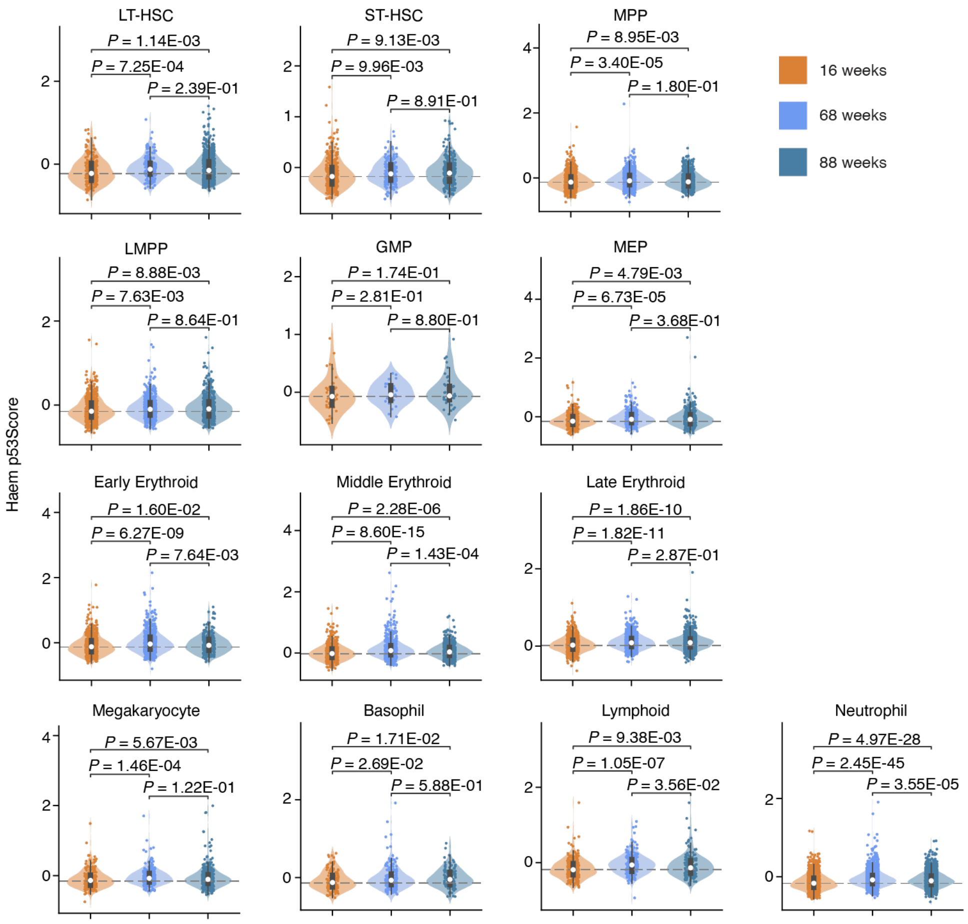


**Figure S7, relating to Figure 6. Elevated p53 activity in aged WT HSPCs**

Comparison of Haem p53Score in scRNA-seq derived transcriptomes of different HSPC types from a 16-week-old and a 68-week-old WT mouse, and an 88-week-old *Fancd2*$^{+/-}$ mouse.

**Table S1, relating to Figure 1.** Top 100 up- and downregulated genes in *Aldh2*$^{-/-}$ *Fancd2*$^{-/-}$ compared to WT LKS. Aldh2 gene is shown but not included in any downstream analysis.

| UPREGULATED | | | DOWNREGULATED | | |
|---|---|---|---|---|---|
| Gene | Log2 fold | FDR | Gene | Log2 fold | FDR |
| *Epha2* | 5.17 | 6.55E-133 | *Aldh2* | -6.19 | 0 |
| *Hmcn2* | 4.43 | 5.05E-121 | *Thbs1* | -6.00 | 2.26E-78 |
| *Gm13067* | 3.17 | 1.52E-87 | *Ncam1* | -3.51 | 2.22E-64 |
| *Dnm3* | 2.27 | 1.17E-86 | *Fxyd1* | -3.15 | 2.94E-62 |
| *Serpine2* | 4.26 | 1.87E-86 | *Pgr* | -4.76 | 1.11E-59 |
| *Phlda3* | 4.50 | 7.11E-82 | *Cdh15* | -2.61 | 2.33E-58 |
| *Cpt1c* | 3.56 | 3.04E-75 | *Dmc1* | -2.25 | 9.54E-53 |
| *Etv4* | 3.59 | 2.09E-72 | *Iglc2* | -1.89 | 1.63E-47 |
| *Ms4a3* | 2.73 | 1.39E-68 | *Htra3* | -2.88 | 2.45E-46 |
| *Nrp1* | 3.77 | 3.88E-65 | *Zfp354a* | -2.63 | 1.35E-45 |
| *Sulf2* | 4.08 | 5.80E-64 | *Fscn1* | -2.92 | 3.62E-42 |
| *Gdf15* | 2.84 | 7.06E-64 | *Fyb2* | -3.36 | 2.40E-41 |
| *Fam212b* | 3.21 | 4.12E-61 | *Cux1* | -0.82 | 6.57E-41 |
| *Ahnak2* | 3.25 | 4.66E-61 | *Bace2* | -2.82 | 1.26E-40 |
| *Cdkn1a* | 3.82 | 4.45E-56 | *Sdk1* | -1.50 | 9.47E-40 |
| *Alox5* | 2.45 | 6.85E-55 | *Medag* | -2.45 | 2.71E-39 |
| *Fabp4* | 3.30 | 5.26E-54 | *AC149090.1* | -0.71 | 7.10E-39 |
| *Eda2r* | 3.66 | 6.40E-53 | *Ndn* | -3.50 | 3.71E-37 |
| *Gem* | 3.47 | 1.18E-52 | *Mpl* | -1.70 | 1.39E-34 |
| *Zmat3* | 3.50 | 4.21E-50 | *Ccl3* | -2.60 | 2.23E-34 |
| *Tjp1* | 3.09 | 1.51E-48 | *Samd5* | -2.18 | 3.88E-34 |
| *Tnfsf4* | 3.28 | 3.29E-48 | *Snrpn* | -1.97 | 3.11E-31 |
| *Lifr* | 2.02 | 2.88E-46 | *Arhgef28* | -3.38 | 5.47E-31 |
| *Gimap4* | 3.23 | 9.25E-44 | *Meg3* | -1.57 | 9.52E-31 |
| *Lgals1* | 2.38 | 2.28E-41 | *Dlk1* | -1.90 | 1.91E-30 |
| *Cd200r4* | 1.52 | 1.05E-40 | *Trpc6* | -2.46 | 4.72E-30 |
| *Ripor3* | 1.73 | 3.18E-40 | *Gm15990* | -1.85 | 1.66E-29 |
| *S100a6* | 2.64 | 6.13E-40 | *Mmrn1* | -2.55 | 1.83E-29 |
| *Rhbdl2* | 2.17 | 1.30E-39 | *Creb5* | -3.05 | 2.00E-29 |
| *Stxbp6* | 2.40 | 4.19E-39 | *Ighd* | -1.22 | 3.17E-29 |
| *Mcpt8* | 2.04 | 9.78E-39 | *Obsl1* | -2.74 | 3.37E-29 |
| *Gm16587* | 1.41 | 1.76E-38 | *Gm42517* | -1.86 | 2.09E-28 |
| *Rasal1* | 2.02 | 5.63E-37 | *Smoc1* | -2.93 | 5.23E-28 |
| *Slamf1* | 2.38 | 2.91E-36 | *Serpinf1* | -2.49 | 1.31E-27 |
| *Spry4* | 2.54 | 4.59E-36 | *Myl10* | -1.96 | 8.53E-27 |
| *BC049352* | 2.49 | 4.95E-35 | *Rftn2* | -2.86 | 3.05E-26 |
| *Clca3a1* | 1.92 | 1.01E-34 | *P2ry10b* | -2.09 | 2.31E-25 |
| *Socs2* | 2.32 | 1.02E-33 | *Srms* | -1.63 | 5.39E-25 |
| *Stab2* | 2.76 | 6.80E-32 | *Rnf208* | -2.58 | 3.69E-24 |
| *Bmp7* | 1.78 | 1.01E-31 | *Obscn* | -2.21 | 3.99E-24 |
| *Selp* | 2.54 | 2.24E-31 | *Itsn1* | -1.72 | 5.28E-24 |
| *Gm3453* | 1.22 | 2.87E-31 | *Zfp697* | -2.19 | 5.96E-24 |
| *C79798* | 1.64 | 1.66E-30 | *Inca1* | -2.08 | 8.22E-24 |
| *Tmem156* | 1.39 | 2.94E-30 | *2610206C17Rik* | -1.41 | 9.37E-24 |
| *Grap* | 2.56 | 4.06E-30 | *Als2cl* | -2.39 | 9.71E-24 |
| *Gm42047* | 2.29 | 6.43E-30 | *Nes* | -1.62 | 1.36E-23 |
| *Cma1* | 1.62 | 1.68E-29 | *Dnah7b* | -1.46 | 1.40E-23 |
| *Tmsb4x* | 0.52 | 1.84E-29 | *Arhgap8* | -1.49 | 2.68E-23 |
| *Crip2* | 2.65 | 7.75E-29 | *Trpv4* | -1.78 | 6.84E-23 |
| *Pmaip1* | 1.98 | 1.34E-28 | *Aire* | -2.00 | 1.08E-22 |
| *C130083M11Rik* | 1.55 | 1.99E-28 | *Shisa9* | -1.30 | 1.09E-22 |
| *Chga* | 2.55 | 2.70E-28 | *Flt3* | -1.38 | 1.26E-22 |

| | | | | | |
|---|---|---|---|---|---|
| *Sel1l3* | 1.90 | 5.28E-28 | *Hcn3* | -2.55 | 1.51E-22 |
| *Ccr1* | 2.37 | 2.64E-27 | *Airn* | -2.33 | 2.93E-22 |
| *Ccr5* | 2.15 | 1.28E-26 | *Abcg3* | -2.35 | 3.20E-22 |
| *Chst2* | 2.06 | 1.68E-26 | *Fam161a* | -2.15 | 7.96E-22 |
| *Ier5l* | 1.86 | 1.68E-26 | *AC139671.1* | -1.85 | 1.33E-21 |
| *Thy1* | 1.82 | 2.12E-26 | *Slc5a9* | -2.02 | 1.36E-21 |
| *Dglucy* | 2.58 | 2.57E-26 | *Gm19590* | -1.19 | 1.57E-21 |
| *Plk2* | 2.04 | 4.03E-26 | *Gpc3* | -1.97 | 2.12E-21 |
| *Fam20a* | 1.79 | 9.74E-26 | *Paqr5* | -2.26 | 4.16E-21 |
| *Pvt1* | 2.34 | 3.13E-25 | *Wdr86* | -2.31 | 1.99E-20 |
| *Plpp1* | 2.23 | 4.23E-25 | *Gm10419* | -1.98 | 3.39E-20 |
| *Crygn* | 1.35 | 9.80E-25 | *Clmn* | -2.32 | 5.00E-20 |
| *Hdc* | 1.17 | 1.24E-24 | *Ust* | -1.94 | 9.05E-20 |
| *Tnfsf8* | 2.26 | 1.36E-24 | *Itgb5* | -2.37 | 1.45E-19 |
| *Fcgr3* | 2.39 | 1.38E-24 | *Slc22a4* | -1.87 | 2.28E-19 |
| *Phf11d* | 2.21 | 2.64E-24 | *Ern1* | -1.15 | 3.63E-19 |
| *Plvap* | 2.58 | 3.68E-24 | *Boll* | -1.38 | 5.03E-19 |
| *Ptgir* | 2.51 | 5.83E-24 | *Tgfa* | -1.67 | 5.82E-19 |
| *Nkg7* | 1.57 | 6.96E-24 | *Fxyd7* | -0.85 | 6.21E-19 |
| *Cxcr1* | 2.08 | 8.11E-24 | *Hlf* | -0.96 | 7.51E-19 |
| *Nuak1* | 1.65 | 1.35E-23 | *Adgrg1* | -0.52 | 9.07E-19 |
| *Ccnd1* | 1.92 | 2.62E-23 | *Rprm* | -1.22 | 1.03E-18 |
| *Vcam1* | 2.24 | 4.27E-23 | *Gm47270* | -1.49 | 1.06E-18 |
| *Sox18* | 2.13 | 5.62E-23 | *Plag1* | -2.23 | 1.14E-18 |
| *Gpr182* | 2.04 | 5.68E-23 | *Wdr54* | -1.61 | 2.81E-18 |
| *Tpsb2* | 1.48 | 1.02E-22 | *Ptn* | -1.38 | 3.46E-18 |
| *Rgs8* | 1.53 | 1.09E-22 | *Dnah1* | -2.29 | 7.09E-18 |
| *Gdpd5* | 1.71 | 1.40E-22 | *Slc35d3* | -2.05 | 8.87E-18 |
| *Sh3gl3* | 1.58 | 2.63E-22 | *Pim1* | -0.92 | 1.63E-17 |
| *Fbn1* | 2.20 | 3.92E-22 | *Iqgap2* | -0.78 | 1.85E-17 |
| *Igfbp7* | 2.27 | 6.16E-22 | *6430573P05Rik* | -1.42 | 2.38E-17 |
| *Pde10a* | 0.88 | 1.11E-21 | *Gm42439* | -1.13 | 2.94E-17 |
| *Gtse1* | 1.76 | 1.82E-20 | *Ddx5* | -0.30 | 4.19E-17 |
| *Rps27l* | 0.79 | 2.95E-20 | *Spag6l* | -1.62 | 4.46E-17 |
| *Espnl* | 1.53 | 5.33E-20 | *Greb1* | -2.26 | 5.22E-17 |
| *Adgrg5* | 1.69 | 5.55E-20 | *Klrb1a* | -1.66 | 6.84E-17 |
| *Itga1* | 2.00 | 7.76E-20 | *Ifitm1* | -0.86 | 8.33E-17 |
| *Cldn5* | 1.90 | 1.74E-19 | *Slc16a12* | -2.01 | 1.14E-16 |
| *Mcpt4* | 1.29 | 1.88E-19 | *1700040D17Rik* | -1.41 | 1.56E-16 |
| *Clec10a* | 1.80 | 2.31E-19 | *Gsto2* | -1.14 | 1.86E-16 |
| *Cd48* | 1.35 | 3.39E-19 | *Epha7* | -2.22 | 2.28E-16 |
| *Gm16341* | 1.04 | 6.35E-19 | *Kbtbd12* | -1.80 | 4.64E-16 |
| *Synm* | 1.62 | 1.06E-18 | *Zfp300* | -1.95 | 4.87E-16 |
| *6430548M08Rik* | 1.73 | 1.23E-18 | *4921504A21Rik* | -1.36 | 5.03E-16 |
| *4930519L02Rik* | 1.52 | 1.76E-18 | *Ddi1* | -1.08 | 6.01E-16 |
| *Cnn3* | 1.86 | 7.30E-18 | *Ephb4* | -2.04 | 9.15E-16 |
| *Myo1b* | 1.59 | 1.10E-17 | *Itgam* | -1.52 | 9.75E-16 |
| *Notch3* | 2.05 | 1.14E-17 | *Nptxr* | -1.44 | 1.19E-15 |
| | | | *Tmem102* | -2.03 | 1.52E-15 |

**Table S2, relating to Figure 1.** Enriched GO terms of top 100 up- and downregulated genes *Aldh2*$^{-/-}$ *Fancd2*$^{-/-}$ compared to WT LKS.

**GO terms from upregulated genes**

| GO term ID | Term description | Strength | FDR |
|---|---|---|---|
| GO:0060384 | Innervation | 1.54 | 0.0031 |
| GO:0072332 | Intrinsic apoptotic signaling pathway by p53 class mediator | 1.38 | 0.0013 |
| GO:0060326 | Cell chemotaxis | 1.13 | 1.22E-05 |
| GO:0022409 | Positive regulation of cell-cell adhesion | 0.98 | 0.00012 |
| GO:0045665 | Negative regulation of neuron differentiation | 0.87 | 0.0048 |
| GO:0022407 | Regulation of cell-cell adhesion | 0.81 | 0.00073 |
| GO:0045785 | Positive regulation of cell adhesion | 0.8 | 0.00087 |
| GO:0006954 | Inflammatory response | 0.78 | 0.00054 |
| GO:0010721 | Negative regulation of cell development | 0.75 | 0.0081 |
| GO:0030155 | Regulation of cell adhesion | 0.68 | 0.00073 |
| GO:0030335 | Positive regulation of cell migration | 0.67 | 0.0067 |
| GO:0007155 | Cell adhesion | 0.66 | 0.0011 |
| GO:0051093 | Negative regulation of developmental process | 0.64 | 6.37E-05 |
| GO:0010648 | Negative regulation of cell communication | 0.61 | 5.39E-05 |
| GO:0009968 | Negative regulation of signal transduction | 0.61 | 7.33E-05 |
| GO:0045664 | Regulation of neuron differentiation | 0.61 | 0.006 |
| GO:0023057 | Negative regulation of signaling | 0.6 | 5.39E-05 |
| GO:0051241 | Negative regulation of multicellular organismal process | 0.57 | 0.00026 |
| GO:0030334 | Regulation of cell migration | 0.57 | 0.0067 |
| GO:0016477 | Cell migration | 0.57 | 0.0099 |
| GO:0048585 | Negative regulation of response to stimulus | 0.55 | 7.94E-05 |
| GO:0035556 | Intracellular signal transduction | 0.54 | 0.00062 |
| GO:0040011 | Locomotion | 0.52 | 0.0071 |
| GO:0060284 | Regulation of cell development | 0.52 | 0.0089 |
| GO:0042127 | Regulation of cell population proliferation | 0.47 | 0.0039 |
| GO:0006928 | Movement of cell or subcellular component | 0.47 | 0.0099 |
| GO:0051240 | Positive regulation of multicellular organismal process | 0.45 | 0.0028 |
| GO:0009605 | Response to external stimulus | 0.44 | 0.001 |
| GO:0045595 | Regulation of cell differentiation | 0.44 | 0.006 |
| GO:0019220 | Regulation of phosphate metabolic process | 0.44 | 0.008 |
| GO:0070887 | Cellular response to chemical stimulus | 0.43 | 0.00054 |
| GO:2000026 | Regulation of multicellular organismal development | 0.43 | 0.0019 |
| GO:0050790 | Regulation of catalytic activity | 0.42 | 0.0028 |
| GO:0007165 | Signal transduction | 0.41 | 1.60E-05 |
| GO:0050793 | Regulation of developmental process | 0.41 | 0.001 |
| GO:0051239 | Regulation of multicellular organismal process | 0.37 | 0.0012 |
| GO:0048523 | Negative regulation of cellular process | 0.33 | 0.00026 |
| GO:0048583 | Regulation of response to stimulus | 0.33 | 0.0045 |
| GO:0051716 | Cellular response to stimulus | 0.32 | 6.37E-05 |
| GO:0042221 | Response to chemical | 0.32 | 0.006 |
| GO:0048519 | Negative regulation of biological process | 0.3 | 0.00073 |
| GO:0050896 | Response to stimulus | 0.29 | 4.71E-05 |
| GO:0048522 | Positive regulation of cellular process | 0.29 | 0.00054 |
| GO:0048518 | Positive regulation of biological process | 0.27 | 0.0012 |
| GO:0032501 | Multicellular organismal process | 0.24 | 0.006 |
| GO:0050794 | Regulation of cellular process | 0.18 | 0.0073 |
| GO:0065007 | Biological regulation | 0.16 | 0.0082 |

**GO terms from downregulated genes**

| GO term ID | Term description | Strength | FDR |
|---|---|---|---|

| | | | |
|---|---|---|---|
| GO:0042127 | Regulation of cell population proliferation | 0.49 | 0.008 |
| GO:0045595 | Regulation of cell differentiation | 0.45 | 0.0094 |
| GO:2000026 | Regulation of multicellular organismal development | 0.43 | 0.009 |
| GO:0048869 | Cellular developmental process | 0.41 | 6.82E-05 |
| GO:0030154 | Cell differentiation | 0.4 | 0.00031 |
| GO:0050793 | Regulation of developmental process | 0.39 | 0.0092 |
| GO:0007275 | Multicellular organism development | 0.32 | 0.0014 |
| GO:0048856 | Anatomical structure development | 0.31 | 0.0021 |
| GO:0048731 | System development | 0.31 | 0.0094 |
| GO:0032502 | Developmental process | 0.29 | 0.0033 |
| GO:0048522 | Positive regulation of cellular process | 0.28 | 0.0067 |
| GO:0050794 | Regulation of cellular process | 0.2 | 0.0031 |
| GO:0065007 | Biological regulation | 0.18 | 0.0065 |

**Table S3, relating to Figure 1.** Enriched GO terms of top 100 upregulated genes in respective HSPC cell types from *Aldh2*$^{-/-}$ *Fancd2*$^{-/-}$ compared to WT mice.

**GO terms from upregulated genes in LT-HSC**

| GO term ID | Term description | Strength | FDR |
|---|---|---|---|
| GO:0043129 | Surfactant homeostasis | 1.7 | 0.0109 |
| GO:0035584 | Calcium-mediated signaling using intracellular calcium source | 1.52 | 0.0229 |
| GO:0019934 | cGMP-mediated signaling | 1.5 | 0.0249 |
| GO:0001945 | Lymph vessel development | 1.44 | 0.0311 |
| GO:0048041 | Focal adhesion assembly | 1.41 | 0.0361 |
| GO:0043534 | Blood vessel endothelial cell migration | 1.34 | 0.0491 |
| GO:0051893 | Regulation of focal adhesion assembly | 1.19 | 0.0249 |
| GO:0043542 | Endothelial cell migration | 1.18 | 0.0265 |
| GO:0030193 | Regulation of blood coagulation | 1.17 | 0.0087 |
| GO:0097755 | Positive regulation of blood vessel diameter | 1.11 | 0.0392 |
| GO:0007160 | Cell-matrix adhesion | 1.09 | 0.0043 |
| GO:0008643 | Carbohydrate transport | 1.09 | 0.0428 |
| GO:0019395 | Fatty acid oxidation | 1.08 | 0.0454 |
| GO:0030879 | Mammary gland development | 1.01 | 0.003 |
| GO:0061041 | Regulation of wound healing | 1.01 | 0.009 |
| GO:0048593 | Camera-type eye morphogenesis | 1 | 0.0095 |
| GO:0001952 | Regulation of cell-matrix adhesion | 0.99 | 0.0273 |
| GO:0048660 | Regulation of smooth muscle cell proliferation | 0.92 | 0.017 |
| GO:0045765 | Regulation of angiogenesis | 0.87 | 0.00097 |
| GO:0001525 | Angiogenesis | 0.85 | 0.0012 |
| GO:0003018 | Vascular process in circulatory system | 0.83 | 0.0365 |
| GO:1901888 | Regulation of cell junction assembly | 0.82 | 0.0406 |
| GO:0010810 | Regulation of cell-substrate adhesion | 0.82 | 0.0428 |
| GO:0001944 | Vasculature development | 0.79 | 9.79E-05 |
| GO:0001568 | Blood vessel development | 0.78 | 0.00017 |
| GO:0003007 | Heart morphogenesis | 0.78 | 0.0258 |
| GO:0048514 | Blood vessel morphogenesis | 0.77 | 0.0019 |
| GO:0098609 | Cell-cell adhesion | 0.77 | 0.0037 |
| GO:0019932 | Second-messenger-mediated signaling | 0.77 | 0.0146 |
| GO:0048871 | Multicellular organismal homeostasis | 0.76 | 0.0165 |

**GO terms from upregulated genes in MPP**

| GO term ID | Term description | Strength | FDR |
|---|---|---|---|
| GO:0048295 | Positive regulation of isotype switching to ige isotypes | 2.11 | 0.0413 |
| GO:0060979 | Vasculogenesis involved in coronary vascular morphogenesis | 1.69 | 0.0225 |
| GO:0090050 | Positive regulation of cell migration involved in sprouting angiogenesis | 1.57 | 0.032 |
| GO:0060977 | Coronary vasculature morphogenesis | 1.52 | 0.0092 |
| GO:0060384 | Innervation | 1.44 | 0.0419 |
| GO:2000515 | Negative regulation of cd4-positive, alpha-beta t cell activation | 1.4 | 0.0478 |
| GO:1903672 | Positive regulation of sprouting angiogenesis | 1.36 | 0.0211 |
| GO:0055010 | Ventricular cardiac muscle tissue morphogenesis | 1.3 | 0.0266 |
| GO:0043536 | Positive regulation of blood vessel endothelial cell migration | 1.25 | 0.0304 |
| GO:0003208 | Cardiac ventricle morphogenesis | 1.23 | 0.0115 |
| GO:0061180 | Mammary gland epithelium development | 1.2 | 0.0356 |
| GO:0043535 | Regulation of blood vessel endothelial cell migration | 1.14 | 0.0211 |
| GO:0003206 | Cardiac chamber morphogenesis | 1.05 | 0.0133 |
| GO:0045766 | Positive regulation of angiogenesis | 0.93 | 0.0296 |
| GO:0030308 | Negative regulation of cell growth | 0.9 | 0.0356 |
| GO:0060326 | Cell chemotaxis | 0.9 | 0.0356 |
| GO:0010632 | Regulation of epithelial cell migration | 0.89 | 0.0211 |
| GO:0022409 | Positive regulation of cell-cell adhesion | 0.85 | 0.0266 |
| GO:0045926 | Negative regulation of growth | 0.84 | 0.0294 |
| GO:0032103 | Positive regulation of response to external stimulus | 0.77 | 0.0173 |

| | | | |
|---|---|---|---|
| GO:0048732 | Gland development | 0.76 | 0.0049 |
| GO:0045765 | Regulation of angiogenesis | 0.76 | 0.0419 |
| GO:0022407 | Regulation of cell-cell adhesion | 0.7 | 0.0413 |
| GO:0045785 | Positive regulation of cell adhesion | 0.69 | 0.0435 |
| GO:0006935 | Chemotaxis | 0.68 | 0.0342 |
| GO:0001558 | Regulation of cell growth | 0.68 | 0.0451 |
| GO:0051347 | Positive regulation of transferase activity | 0.61 | 0.0408 |
| GO:0048729 | Tissue morphogenesis | 0.6 | 0.041 |
| GO:0051962 | Positive regulation of nervous system development | 0.6 | 0.0413 |
| GO:0045597 | Positive regulation of cell differentiation | 0.58 | 0.0056 |

**GO terms from upregulated genes in LMPP**

| GO term ID | Term description | Strength | FDR |
|---|---|---|---|
| GO:0022409 | Positive regulation of cell-cell adhesion | 0.94 | 0.0323 |
| GO:0022407 | Regulation of cell-cell adhesion | 0.84 | 0.0267 |
| GO:0030155 | Regulation of cell adhesion | 0.7 | 0.0323 |
| GO:0051338 | Regulation of transferase activity | 0.61 | 0.0439 |
| GO:0051128 | Regulation of cellular component organization | 0.42 | 0.0439 |
| GO:0009966 | Regulation of signal transduction | 0.4 | 0.0375 |
| GO:0010646 | Regulation of cell communication | 0.38 | 0.0375 |
| GO:0023051 | Regulation of signaling | 0.38 | 0.0375 |
| GO:0048523 | Negative regulation of cellular process | 0.33 | 0.0293 |
| GO:0048522 | Positive regulation of cellular process | 0.32 | 0.0244 |
| GO:0048518 | Positive regulation of biological process | 0.3 | 0.0244 |
| GO:0050896 | Response to stimulus | 0.26 | 0.0323 |

**GO terms from upregulated genes in MEP**

| GO term ID | Term description | Strength | FDR |
|---|---|---|---|
| GO:0072332 | Intrinsic apoptotic signaling pathway by p53 class mediator | 1.52 | 0.00069 |
| GO:0097193 | Intrinsic apoptotic signaling pathway | 1.17 | 0.00069 |
| GO:0008630 | Intrinsic apoptotic signaling pathway in response to dna damage | 1.35 | 0.0019 |
| GO:0097190 | Apoptotic signaling pathway | 0.96 | 0.0021 |
| GO:0007165 | Signal transduction | 0.36 | 0.0062 |
| GO:0023052 | Signaling | 0.34 | 0.0082 |
| GO:0007154 | Cell communication | 0.33 | 0.0108 |
| GO:0042771 | Intrinsic apoptotic signaling pathway in response to dna damage by p53 class mediator | 1.56 | 0.0108 |
| GO:0050896 | Response to stimulus | 0.25 | 0.0108 |
| GO:0006935 | Chemotaxis | 0.76 | 0.0126 |
| GO:0040011 | Locomotion | 0.55 | 0.0188 |
| GO:0051716 | Cellular response to stimulus | 0.27 | 0.0194 |
| GO:0035556 | Intracellular signal transduction | 0.5 | 0.0295 |
| GO:0006950 | Response to stress | 0.36 | 0.0325 |

**GO terms from upregulated genes in GMP**

| GO term ID | Term description | Strength | FDR |
|---|---|---|---|
| GO:0002438 | Acute inflammatory response to antigenic stimulus | 1.77 | 0.0443 |
| GO:0002526 | Acute inflammatory response | 1.25 | 0.0257 |
| GO:0032101 | Regulation of response to external stimulus | 0.64 | 0.0105 |
| GO:0009968 | Negative regulation of signal transduction | 0.59 | 0.0105 |
| GO:0010648 | Negative regulation of cell communication | 0.57 | 0.0105 |
| GO:0023057 | Negative regulation of signaling | 0.57 | 0.0105 |
| GO:0048585 | Negative regulation of response to stimulus | 0.54 | 0.0105 |
| GO:0048583 | Regulation of response to stimulus | 0.33 | 0.0378 |
| GO:0050896 | Response to stimulus | 0.24 | 0.029 |

**Table S4, relating to Figure 5.** Clinical characteristics of TCGA AML cases with low and high Haem p53Score. For ELN 2022 risk stratification, AML cases with PML-RARA translocation were included in the favourable category. Statistical testing of significance for median age and mean bone marrow blast % were calculated using two-tailed unpaired t test. All other statistical testing in this table used Chi-square test.

| | | p53Score low (n = 102) | | p53Score high (n = 36) | | p value |
|---|---|---|---|---|---|---|
| | | Number | % | Number | % | |
| Age | Median + (range) | 51 | (21 - 77) | 61 | (31 - 88) | 0.001 |
| | <60 | 66 | 59 | 16 | 44 | 0.033 |
| | >60 | 36 | 41 | 20 | 56 | |
| Gender | Female | 46 | 45 | 16 | 44 | 0.946 |
| | Male | 56 | 55 | 20 | 56 | |
| Prior treatment | Yes | 22 | 22 | 16 | 44 | 0.008 |
| | No | 80 | 78 | 20 | 56 | |
| Prior malignancy | Yes | 7 | 7 | 3 | 8 | 0.77 |
| | No | 95 | 93 | 33 | 92 | |
| Race | White | 86 | 84 | 36 | 100 | 0.094 |
| | Black or african american | 13 | 13 | 0 | 0 | |
| | Asian | 2 | 2 | 0 | 0 | |
| | Not reported | 1 | 1 | 0 | 0 | |
| Bone marrow blast % | Mean + (range) | 69 | (30 - 100) | 70 | (30 - 98) | 0.86 |
| ELN 2022 Risk | Favourable | 23 | 23 | 13 | 36 | 0.236 |
| | Intermediate | 41 | 41 | 14 | 39 | |
| | Adverse | 37 | 36 | 9 | 25 | |

**Table S5, relating to Figure 5.** Somatic driver mutations in TCGA AML cases with low and high Haem p53Score. Class 2 mutations consist of *PML-RARA*, *MYH11-CBFB*, *RUNX1-RUNX1T1*, *MLL* rearrangement translocations, *RUNX1* and *CEBPA* mutations. Statistical significance tested by Chi-square test.

| Somatic driver mutations | | p53Score low (102) | | p53Score high (36) | | p value |
|---|---|---|---|---|---|---|
| | | Number | % | Number | % | |
| *PML-RARA* | Positive | 13 | 11 | 2 | 5 | 0.289 |
| | Negative | 101 | 89 | 35 | 95 | |
| *MYH11-CBFB* | Positive | 5 | 4 | 5 | 14 | 0.052 |
| | Negative | 109 | 96 | 32 | 86 | |
| *RUNX1-RUNX1T1* | Positive | 7 | 6 | 0 | 0 | 0.123 |
| | Negative | 107 | 94 | 37 | 100 | |
| *MLL* rearrangements | Positive | 6 | 5 | 2 | 5 | 0.973 |
| | Negative | 108 | 95 | 35 | 95 | |
| *BCR-ABL1* | Positive | 4 | 3.5 | 0 | 0 | 0.248 |
| | Negative | 110 | 96 | 37 | 100 | |
| *NUP98-NSD1* | Positive | 1 | 1 | 2 | 5 | 0.086 |
| | Negative | 113 | 99 | 35 | 95 | |
| *NPM1* | Positive | 26 | 23 | 14 | 38 | 0.072 |
| | Negative | 88 | 77 | 23 | 62 | |
| *FLT3-ITD* | Positive | 28 | 25 | 10 | 27 | 0.764 |
| | Negative | 86 | 75 | 27 | 73 | |
| *MLL-PTD* | Positive | 5 | 4 | 2 | 5 | 0.798 |
| | Negative | 109 | 96 | 35 | 95 | |
| *FLT3* | Positive | 7 | 6 | 3 | 8 | 0.676 |
| | Negative | 107 | 94 | 34 | 92 | |
| *CEBPA* | Positive | 15 | 13 | 2 | 5 | 0.195 |
| | Negative | 99 | 87 | 35 | 95 | |
| *DNMT3A* | Positive | 27 | 24 | 9 | 24 | 0.937 |
| | Negative | 87 | 76 | 28 | 76 | |
| *IDH1* | Positive | 12 | 11 | 1 | 3 | 0.14 |
| | Negative | 102 | 89 | 36 | 97 | |
| *IDH2* | Positive | 13 | 11 | 4 | 11 | 0.921 |
| | Negative | 101 | 89 | 33 | 89 | |
| *RUNX1* | Positive | 17 | 15 | 1 | 3 | 0.046 |
| | Negative | 97 | 85 | 36 | 97 | |
| *TP53* | Positive | 12 | 11 | 1 | 3 | 0.14 |

| | | | | | | |
|---|---|---|---|---|---|---|
| | Negative | 102 | 89 | 36 | 97 | |
| *TET2* | Positive | 12 | 11 | 3 | 8 | 0.669 |
| | Negative | 102 | 89 | 34 | 92 | |
| *WT1* | Positive | 10 | 9 | 2 | 5 | 0.511 |
| | Negative | 104 | 91 | 35 | 95 | |
| *BCOR* | Positive | 2 | 2 | 0 | 0 | 0.417 |
| | Negative | 112 | 98 | 37 | 100 | |
| *ASXL1* | Positive | 7 | 6 | 0 | 0 | 0.123 |
| | Negative | 107 | 94 | 37 | 100 | |
| *SRSF2* | Positive | 10 | 9 | 2 | 5 | 0.511 |
| | Negative | 104 | 91 | 35 | 95 | |
| *SF3B1* | Positive | 4 | 3 | 1 | 97 | 0.812 |
| | Negative | 110 | 97 | 36 | 3 | |
| *U2AF1* | Positive | 4 | 4 | 3 | 8 | 0.248 |
| | Negative | 110 | 96 | 34 | 92 | |
| *RUNX1, RUNX1-RUNX1T1* | Positive | 24 | 21 | 1 | 3 | 0.009 |
| | Negative | 90 | 79 | 36 | 97 | |
| *RUNX1, RUNX1-RUNX1T1 CEPBA* | Positive | 39 | 34 | 3 | 8 | 0.002 |
| | Negative | 75 | 66 | 34 | 92 | |
| CLASS 2 | Positive | 62 | 54 | 12 | 32 | 0.02 |
| | Negative | 52 | 46 | 25 | 68 | |

**Table S6, relating to Figure 5.** Pearson coefficient (>0.1) of correlation between gene expression and Haem p53Score in LKS transcriptomes of *Aldh2$^{-/-}$ Fancd2$^{-/-}$*.

| Gene | Pearson | Gene | Pearson | Gene | Pearson |
|---|---|---|---|---|---|
| *Phlda3* | 0.64 | *Reep2* | 0.15 | *Gm8186* | 0.13 |
| *Zmat3* | 0.60 | *Dcxr* | 0.15 | *Mdm2* | 0.13 |
| *Eda2r* | 0.60 | *Itgb2* | 0.15 | *Rrm2* | 0.12 |
| *Cdkn1a* | 0.59 | *Gadl1* | 0.15 | *Fam120a* | 0.12 |
| *Pvt1* | 0.53 | *Ssbp4* | 0.15 | *Rps26* | 0.12 |
| *Gm13067* | 0.50 | *Glipr1* | 0.15 | *Slc6a13* | 0.12 |
| *Sulf2* | 0.48 | *Igsf9b* | 0.15 | *Uqcrq* | 0.12 |
| *Epha2* | 0.48 | *Rps19* | 0.15 | *Galnt6* | 0.12 |
| *Serpine2* | 0.47 | *Eif5a* | 0.15 | *Naa20* | 0.12 |
| *Hmcn2* | 0.46 | *Lacc1* | 0.15 | *Nedd8* | 0.12 |
| *Tnfsf4* | 0.43 | *Arap3* | 0.14 | *Glrx3* | 0.12 |
| *Bax* | 0.43 | *Mt1* | 0.14 | *Thy1* | 0.12 |
| *Cpt1c* | 0.43 | *Arpc2* | 0.14 | *Cox6b1* | 0.12 |
| *Fam212b* | 0.42 | *F2r* | 0.14 | *Cenpw* | 0.12 |
| *Bbc3* | 0.40 | *Hprt* | 0.14 | *Trpm2* | 0.12 |
| *Ccng1* | 0.39 | *Fam198b* | 0.14 | *Ube2k* | 0.12 |
| *Gm42047* | 0.36 | *Nme1* | 0.14 | *Gm4707* | 0.12 |
| *Aen* | 0.36 | *Uqcr10* | 0.14 | *Myh9* | 0.12 |
| *Tnfrsf10b* | 0.35 | *Rhbdl2* | 0.14 | *Dapk1* | 0.12 |
| *Perp* | 0.33 | *Gm10076* | 0.14 | *Enc1* | 0.12 |
| *Dglucy* | 0.32 | *Naip2* | 0.14 | *Hmga1b* | 0.12 |
| *Rps27l* | 0.32 | *Commd3* | 0.14 | *Snx4* | 0.12 |
| *S100a6* | 0.30 | *Sem1* | 0.14 | *Sod2* | 0.12 |
| *Cox6b2* | 0.29 | *Gpr68* | 0.14 | *Crip2* | 0.12 |
| *Selp* | 0.29 | *Ybx3* | 0.14 | *Rapgef3* | 0.12 |
| *Tjp1* | 0.29 | *E2f1* | 0.14 | *Ctsg* | 0.12 |
| *Lgals1* | 0.28 | *Frrs1* | 0.14 | *Ndufb7* | 0.12 |
| *Ier5* | 0.27 | *Eif2s1* | 0.14 | *Mcm2* | 0.12 |
| *Plk2* | 0.27 | *Cd9* | 0.14 | *Gm3453* | 0.12 |
| *Psrc1* | 0.26 | *Ss18l2* | 0.14 | *Dusp3* | 0.12 |
| *Gdf15* | 0.25 | *Exoc4* | 0.14 | *Spred2* | 0.12 |
| *Slc19a2* | 0.25 | *Rpl8* | 0.14 | *Ak6* | 0.12 |
| *Pmaip1* | 0.25 | *Ndufb6* | 0.14 | *Pla2g4a* | 0.12 |
| *Pfn1* | 0.24 | *Gstm2* | 0.14 | *Mpp4* | 0.12 |
| *Etv4* | 0.24 | *Gfer* | 0.14 | *Snrpd1* | 0.12 |
| *Sesn2* | 0.23 | *Psma2* | 0.14 | *Ccnb2* | 0.12 |
| *Cd55* | 0.22 | *Micall2* | 0.14 | *Myl12a* | 0.12 |
| *Trp53cor1* | 0.22 | *Cdc34b* | 0.14 | *Sdsl* | 0.12 |
| *Emilin2* | 0.22 | *Cox6a1* | 0.14 | *C1qbp* | 0.12 |
| *Pml* | 0.22 | *Sdf2l1* | 0.14 | *Bend4* | 0.12 |
| *Gtse1* | 0.22 | *Tab2* | 0.14 | *Vwf* | 0.12 |
| *Ctsd* | 0.22 | *Rpl32* | 0.14 | *Clec4e* | 0.12 |
| *Slamf1* | 0.22 | *Snrpf* | 0.14 | *Serbp1* | 0.12 |
| *Tuba8* | 0.21 | *Smdt1* | 0.14 | *Thyn1* | 0.12 |
| *Lgals9* | 0.21 | *Cox6c* | 0.14 | *Ndufv2* | 0.12 |
| *Ms4a3* | 0.21 | *Atp5j* | 0.14 | *Tomm7* | 0.12 |
| *Etfb* | 0.21 | *Ndufa1* | 0.14 | *Tipin* | 0.12 |
| *4930519L02* | 0.21 | *S100a10* | 0.14 | *Cep170b* | 0.12 |
| *Ces2g* | 0.21 | *C530008M1* | 0.14 | *Ccr1* | 0.12 |
| *Casp1* | 0.20 | *Ptpn7* | 0.14 | *Mrpl12* | 0.12 |
| *Tgfbr3* | 0.20 | *Atp5e* | 0.14 | *Ddit4* | 0.12 |
| *Nkg7* | 0.20 | *Gm10131* | 0.14 | *Usmg5* | 0.12 |
| *Tmsb4x* | 0.20 | *Dnm3* | 0.14 | *Psma5* | 0.12 |
| *C130083M1* | 0.20 | *Podxl* | 0.14 | *Hnrnpc* | 0.12 |
| *Trp53inp1* | 0.20 | *Map3k20* | 0.14 | *Rpl13a-ps1* | 0.12 |
| *Cfl1* | 0.19 | *Minos1* | 0.14 | *Gm4950* | 0.12 |
| *P2ry1* | 0.19 | *Uqcrb* | 0.14 | *Ptbp3* | 0.12 |

| | | | | | |
|---|---|---|---|---|---|
| *Unc119* | *0.19* | *Gch1* | *0.14* | *Col18a1* | *0.12* |
| *Pak6* | *0.19* | *Vill* | *0.14* | *Tomm5* | *0.12* |
| *Gm15675* | *0.19* | *Atox1* | *0.13* | *Nhsl2* | *0.12* |
| *Alox5* | *0.19* | *Aaas* | *0.13* | *Ndufa12* | *0.12* |
| *Ccnd1* | *0.19* | *Etf1* | *0.13* | *Magoh* | *0.12* |
| *C1qtnf6* | *0.19* | *Mbnl1* | *0.13* | *Prelid3b* | *0.12* |
| *Ahnak2* | *0.18* | *Actb* | *0.13* | *Stxbp6* | *0.12* |
| *Ass1* | *0.18* | *Ifi47* | *0.13* | *Gt(ROSA)2* | *0.12* |
| *Synm* | *0.18* | *Ndufa13* | *0.13* | *Rpn1* | *0.12* |
| *H2afj* | *0.18* | *Pfdn6* | *0.13* | *Myh10* | *0.12* |
| *Atpif1* | *0.18* | *Hsp90aa1* | *0.13* | *Rnf169* | *0.12* |
| *Calm1* | *0.18* | *Polr1d* | *0.13* | *Psma7* | *0.12* |
| *Pik3r6* | *0.18* | *Glrx5* | *0.13* | *Pomp* | *0.12* |
| *Tgfb1* | *0.18* | *Naip6* | *0.13* | *Slpi* | *0.12* |
| *Pmm1* | *0.17* | *Ccnd2* | *0.13* | *Gp5* | *0.12* |
| *Cox4i1* | *0.17* | *Uqcc2* | *0.13* | *1110008P1* | *0.12* |
| *Fas* | *0.17* | *Abracl* | *0.13* | *Ddx21* | *0.12* |
| *Cdc34* | *0.17* | *Shank3* | *0.13* | *Nrp1* | *0.12* |
| *Ak1* | *0.17* | *Atp5f1* | *0.13* | *Cops9* | *0.12* |
| *Socs2* | *0.17* | *Slc35b1* | *0.13* | *Lama5* | *0.12* |
| *Ldha* | *0.17* | *Aqp1* | *0.13* | *Syce2* | *0.12* |
| *Txn1* | *0.17* | *Rb1* | *0.13* | *Eif4e* | *0.12* |
| *Ppa1* | *0.17* | *Fyn* | *0.13* | *Llph* | *0.11* |
| *Atp5g3* | *0.17* | *Cox5a* | *0.13* | *Phf11d* | *0.11* |
| *Gem* | *0.17* | *Hells* | *0.13* | *Baz1a* | *0.11* |
| *Srgn* | *0.16* | *Polk* | *0.13* | *Id1* | *0.11* |
| *Dubr* | *0.16* | *Slc25a3* | *0.13* | *Mcpt8* | *0.11* |
| *Plxna1* | *0.16* | *Cxcr1* | *0.13* | *Btf3* | *0.11* |
| *Rps11* | *0.16* | *Ybx1* | *0.13* | *Gdf3* | *0.11* |
| *Ddias* | *0.16* | *Pidd1* | *0.13* | *Psmb1* | *0.11* |
| *Ptp4a3* | *0.16* | *Rexo2* | *0.13* | *Als2* | *0.11* |
| *Fcgr3* | *0.16* | *Tma7* | *0.13* | *Klhl22* | *0.11* |
| *Trp53* | *0.16* | *Mrps6* | *0.13* | *Actr3* | *0.11* |
| *Zfp385a* | *0.16* | *Itga2b* | *0.13* | *Grb2* | *0.11* |
| *Cpa3* | *0.16* | *Gm10269* | *0.13* | *Ola1* | *0.11* |
| *Etv5* | *0.16* | *Sec61b* | *0.13* | *Mrps21* | *0.11* |
| *Ecm1* | *0.16* | *Hemgn* | *0.13* | *Ulbp1* | *0.11* |
| *Gata1* | *0.16* | *Siva1* | *0.13* | *Akr1b3* | *0.11* |
| *Ripor3* | *0.16* | *Atp5b* | *0.13* | *Pgk1* | *0.11* |
| *Npm1* | *0.16* | *Ranbp1* | *0.13* | *Ctps* | *0.11* |
| *mt-Nd1* | *0.16* | *Tmem40* | *0.13* | *Dtnbp1* | *0.11* |
| *Dbi* | *0.16* | *Nucks1* | *0.13* | *Calr* | *0.11* |
| *Epor* | *0.16* | *Cox8a* | *0.13* | *Reep5* | *0.11* |
| *Mgmt* | *0.16* | *Gm17087* | *0.13* | *Rpe* | *0.11* |
| *Mfsd2b* | *0.16* | *Arrb2* | *0.13* | *Hnrnpa2b1* | *0.11* |
| *Sh3pxd2b* | *0.16* | *Ndufa11* | *0.13* | *Fth1* | *0.11* |
| *Myo7a* | *0.15* | *Uqcr11* | *0.13* | *Prkd2* | *0.11* |
| *Nudcd2* | *0.15* | *Mob1b* | *0.13* | *Gm6634* | *0.11* |
| *Cyyr1* | *0.15* | *Hdgf* | *0.13* | *Gja1* | *0.11* |
| *2010107E04* | *0.15* | *Hist1h2bc* | *0.13* | *Elob* | *0.11* |
| *Apoe* | *0.15* | *Fgf3* | *0.13* | *Nasp* | *0.11* |
| *Fundc2* | *0.15* | *Fam96a* | *0.13* | *Vamp5* | *0.11* |
| *Apaf1* | *0.15* | *Ei24* | *0.13* | *Ndufa8* | *0.11* |
| *Slc25a5* | *0.15* | *Rpl13a* | *0.13* | *Gpatch4* | *0.11* |
| *Chchd2* | *0.15* | *Grap* | *0.13* | *Srp19* | *0.11* |
| *Cox7b* | *0.15* | *Cox7a2* | *0.13* | *Gm15856* | *0.11* |
| *Dynll1* | *0.15* | *Gimap4* | *0.13* | *Gna15* | *0.11* |
| *Hspe1* | *0.15* | *Phb2* | *0.13* | *Me2* | *0.11* |
| *Cd48* | *0.15* | *Kif5b* | *0.13* | *Metap2* | *0.11* |
| *Dennd2c* | *0.15* | *Notch3* | *0.13* | *Bcl2l1* | *0.11* |
| *Atp5j2* | *0.15* | *Cdc42bpg* | *0.13* | *Tagln2* | *0.11* |
| *Tnk2* | *0.15* | *Degs1* | *0.13* | *Tln1* | *0.11* |

**Table S7, relating to Methods of Haem p53Score.** 694 control genes used to calculate Haem p53Score.

| | | | | | |
|---|---|---|---|---|---|
| *Hspa14* | *Arnt* | *Sept10* | *Tmem199* | *Dnm1l* | *Polr1a* |
| *Sirt4* | *Gpr18* | *Fam69b* | *Zfp619* | *Rpl19* | *Zfp398* |
| *Cacna1a* | *Pde8b* | *Pdcl3* | *Nanos1* | *1810013L24R* | *Hoxaas3* |
| *Gm9844* | *Tspan33* | *Urod* | *Klhdc4* | *Parp12* | *Hspa2* |
| *Taf1c* | *Vnn1* | *Ppa2* | *Fbxl21* | *A830035O19* | *Zfp322a* |
| *Them4* | *Mettl22* | *Slc17a5* | *Gm340* | *Lyst* | *Ptn* |
| *Ermp1* | *Pik3r5* | *Cdt1* | *Zfp143* | *Baiap2* | *Cited2* |
| *Siah1a* | *Snrpb2* | *Grrp1* | *Tomm6* | *Bak1* | *Zfp715* |
| *Cavin1* | *Gm28151* | *Csrp2* | *Gnpda1* | *Arhgap27os3* | *Capn1* |
| *Nupr1l* | *Zfhx2* | *Def8* | *Zfp280c* | *Pde12* | *C330018D20* |
| *Man2b2* | *Kctd15* | *Gm48898* | *4921514A10* | *Morc4* | *Ighv1-12* |
| *Nmd3* | *Cyp20a1* | *Hist1h2ac* | *BC004004* | *Smpd3* | *Afmid* |
| *Insig1* | *Abca8b* | *Eny2* | *D630045J12* | *0610030E20R* | *Hist1h2af* |
| *Dnajb7* | *Vwc2* | *Pms2* | *Qrfp* | *Ptpra* | *Med23* |
| *Sco2* | *Ube4b* | *Nat8f1* | *Smc2* | *Gm11201* | *Pcdhga7* |
| *B2m* | *Fbxo47* | *Mycbp* | *Dffb* | *Gm27206* | *Tnfrsf23* |
| *Kank2* | *Oat* | *Icmt* | *Arid4a* | *Secisbp2* | *Shoc2* |
| *Cln3* | *BC067074* | *Itpripl2* | *Srpk3* | *2610002M06* | *Zfp36* |
| *Gpr21* | *Tbc1d16* | *Tbca* | *Stard13* | *Uvrag* | *Cyfip1* |
| *Fastk* | *Rgl2* | *Rnf215* | *Cldn5* | *Ppp1r26* | *Wdr70* |
| *1700001L05Ri* | *Amfr* | *Gm10501* | *Mcm6* | *Tnfsfm13* | *Gpr45* |
| *Mettl4* | *AC104325.1* | *Nme1* | *Clasp1* | *Piga* | *Dock7* |
| *Thoc7* | *Ddx5* | *Parm1* | *Gm10282* | *Proser1* | *Zfp457* |
| *Tor1aip1* | *Arrdc3* | *Glis2* | *Palm* | *Cse1l* | *Hdx* |
| *9130221H12R* | *Parp10* | *Chst14* | *Cfdp1* | *2310022B05R* | *Cfap97* |
| *Map3k19* | *Tg* | *Gm16755* | *Axin2* | *AC161165.6* | *Agrp* |
| *Zfp532* | *Phldb3* | *Tox4* | *Crebzf* | *Atp10a* | *Hint1* |
| *AC162302.1* | *Hikeshi* | *Gata3* | *Mvb12a* | *Capn5* | *Zcchc17* |
| *Cd276* | *Evc* | *Mbd3* | *Eda* | *Gm49359* | *Prdx3* |
| *Gm26724* | *Faah* | *Map3k9* | *Map1s* | *Edem1* | *Trove2* |
| *9930111J21Ri* | *Smarcb1* | *Igsf8* | *Dtnb* | *Ercc6l* | *Atp6v0a1* |
| *Sytl5* | *Gm46411* | *Mospd3* | *Serpini1* | *Rab10* | *Adck1* |
| *E130308A19R* | *Lrrc40* | *Tefm* | *Mtss1l* | *Kpnb1* | *Grb10* |
| *Irf6* | *Gm37637* | *Slc24a5* | *Ppp1r3f* | *Gm37584* | *C130083M11* |
| *Dlg1* | *Tnf* | *Slc30a4* | *Gm20661* | *Cdr2* | *Gm20681* |
| *Jph1* | *Nr6a1* | *Adgrl2* | *Rhog* | *Gm36445* | *Nup98* |
| *Rpl18a* | *Ppargc1b* | *Zufsp* | *Sec22b* | *Snhg6* | *Gm12258* |
| *Emc7* | *Upf2* | *Rras2* | *Sdha* | *Ywhah* | *Hdac4* |
| *Osbpl3* | *Efr3b* | *Rnase4* | *Pomgnt1* | *Mrps28* | *Crispld2* |
| *Gm44686* | *Gm15563* | *As3mt* | *Gm13561* | *4931403G20* | *Bcs1l* |
| *Itsn1* | *Gm11505* | *Tti2* | *Tctex1d2* | *Gm46515* | *Vwf* |
| *Smtn* | *Ncoa3* | *Srsf3* | *Lsr* | *Mad1l1* | *Eif2s1* |
| *Blm* | *Exoc3l4* | *Spef2* | *Pxk* | *Gm48439* | *Zfp879* |
| *Gm17168* | *Psmg3* | *Hist1h2bh* | *Slc29a3* | *Gm14455* | *Hist1h4k* |
| *Amotl2* | *Oxa1l* | *Terf2ip* | *Cep85l* | *Cradd* | *Gzf1* |
| *Rassf5* | *Nit1* | *Tmco6* | *Armt1* | *Psmd14* | *C1galt1c1* |
| *Gm14302* | *C1qb* | *A330084C13* | *Zbtb39* | *9430015G10* | *Tial1* |
| *Gm14698* | *Arhgap29* | *Npas2* | *Qk* | *Amn1* | *Carns1* |
| *Rela* | *Axin1* | *Hltf* | *Abtb2* | *Tedc2* | *Cacna2d2* |
| *Zbtb20* | *Fam189b* | *Cd74* | *Cant1* | *Esyt3* | *Abca7* |
| *Actb* | *Ccr9* | *Rtf1* | *Soga1* | *Tmem104* | *AC140186.1* |
| *Gm36696* | *Fgd5* | *Hpgd* | *Tmem38a* | *Riok2* | *Sez6l2* |
| *Brms1l* | *Polr1d* | *Junos* | *Rtkn* | *Hspa8* | *Slc31a1* |
| *Gm20406* | *Tldc1* | *Slc2a12* | *Adcy5* | *Smad6* | *Gm17017* |
| *Dnajc27* | *Wdfy4* | *Spout1* | *Fam57a* | *Palld* | *Nudt13* |
| *Gm16024* | *Prkra* | *A530040E14* | *Gm23925* | *Utp6* | *Pard6g* |
| *Gm9888* | *E130307A14* | *Cul7* | *Bpifb5* | *Mpl* | *Dtwd1* |
| *Zfp189* | *Marveld1* | *Lztfl1* | *Upf3a* | *Glb1l* | *Dnmt3bos* |
| *Reep2* | *2610524H06* | *Tle3* | *Ppm1d* | *Prkag2* | *Fmnl2* |

| | | | | | |
|---|---|---|---|---|---|
| Stxbp1 | Rhbdf1 | Hcn3 | Rag1 | Tab3 | Nup85 |
| Evpl | Pabpc4 | Zfp236 | Dync1li1 | Shf | Klhl36 |
| Uba7 | Tln1 | Yae1d1 | Idh2 | Txndc9 | Tbc1d14 |
| Gpkow | 9930012K11R | March3 | Gm10785 | Smpd4 | Usp28 |
| Tmem240 | Slc23a4 | Alyref2 | Spire1 | Fzd7 | Ddb2 |
| Hist1h2bb | Gm48960 | Lzts1 | Ccdc8 | Lamp3 | Ccm2 |
| Mrps31 | Stag3 | AY074887 | Slc31a2 | Gm45890 | Nespas |
| Gm29585 | Gm9958 | Tspan32 | Btbd3 | Oas1g | Rtn4r |
| Nr2f6 | Gatc | Mipep | Pigyl | Mpo | Insig2 |
| Il2 | Cbx2 | Lpin2 | AI854703 | Klhl35 | Gm43518 |
| Gm6563 | Amot | Gm9754 | Dram2 | Rbfa | Mccc1os |
| Vps13d | Pde8a | Paqr3 | Arf4 | Slc17a9 | Padi2 |
| Focad | Vegfc | Hnrnpul2 | Ubtd1 | Poc5 | Vsig10 |
| A430105I19Ri | D830025C05 | Nectin4 | Dbr1 | AC110166.1 | Ranbp6 |
| Hcfc1r1 | Zfp608 | Rhbdl3 | Fli1 | Tnfaip1 | Rin1 |
| Kctd17 | Endov | Ubtd2 | Telo2 | F830016B08 | Drap1 |
| Slc9a5 | Pno1 | Brip1 | Eef1akmt2 | Cpt1a | Uros |
| Ankrd12 | F2rl3 | Zfp647 | Atp6v1e1 | Pex5 | Ttc39c |
| Prdm5 | Tnik | Pwwp2a | Hps1 | Bspry | Arfip1 |
| A930004D18R | Polr3h | Hnrnph2 | Fbxo28 | Cenpo | Tlk1 |
| Arhgef3 | Ivns1abp | Nemp1 | Cyb5r4 | Zcchc2 | Slc38a10 |
| Commd5 | Gdi1 | Naaladl1 | Nat10 | Kcnq5 | Atp6v1a |
| F530104D19R | Angptl7 | Ranbp3 | Cit | Spon1 | Msrb3 |
| Pelp1 | Zfp963 | Kif1bp | Gm16486 | Epb41l3 | Adgra3 |
| Tmem14c | Man2c1os | Gm15996 | Lpin1 | Tnfrsf11a | Speg |
| Rad51 | Arv1 | Hdac9 | Ppme1 | Gm15247 | Rps6 |
| Gm48689 | Syne1 | Pcnx3 | Slc9a6 | Syde2 | Rabep2 |
| Tnni2 | Tacc2 | Cwc15 | Cask | Ppp2r2b | Dennd5b |
| Gm37885 | Exosc4 | Ebp | Wbp1l | Gm40155 | Hsd17b11 |
| Gtf3c3 | Mcoln2 | Hgsnat | Dusp16 | Boc | Pde7b |
| Glipr1 | Zscan29 | Cbx3 | AC163032.1 | Aggf1 | Stx5a |
| Zdhhc14 | Setdb2 | Dnajc30 | Fgf13 | Gm26798 | Ank1 |
| 2810006K23R | 1700056E22R | Ntng2 | Atp1b1 | Pcp2 | Sfxn2 |
| Klf5 | Dnajc21 | Gm47666 | Gm38134 | Cenpj | Ccdc173 |
| Gm29017 | Ap4e1 | Zfp850 | Sult6b1 | Socs6 | Akr7a5 |
| Dnase1 | Spata2 | Otud7b | Etv3 | Gpr146 | |
| Airn | Gm15890 | Gm4489 | Mrps16 | Ubash3b | |
| Dhps | Rhbdl1 | Fbxo16 | Ftl1 | March2 | |
| Ndufa4 | Met | Basp1 | Zfp160 | Mettl7a1 | |
| Zfp84 | Olfr99 | Casp8ap2 | BC022687 | Zpbp | |
| Foxd2 | Ticam1 | D1Pas1 | Esam | Arih2 | |
| Gpsm2 | Hic2 | Rps3a1 | Dnah1 | Gm12355 | |
| Eftud2 | Ube4bos1 | Coq8b | Adamts12 | Ccnyl1 | |
| Clca3a1 | Plekhg5 | Zfp78 | Atxn7 | Foxa3 | |
| Gm44113 | Tnpo3 | Gm553 | Tubgcp3 | Ndnf | |
| Kctd3 | Zdhhc12 | Clp1 | Srgn | Gm45250 | |
| Zmiz2 | Syap1 | Jade3 | Ccser1 | Sh3bp5 | |
| Smarca2 | AW146154 | Sept4 | Mrpl47 | Cnot6 | |
| Usp36 | Hopx | Notch2 | Armc8 | Rhof | |
| Gm15990 | Snrnp25 | Mmaa | 1700110K17 | Aqr | |
| Mill2 | AL732506.1 | Rps3 | Yod1 | Scx | |
| Gm10857 | Saysd1 | Txlna | B3galt2 | Tgfbr1 | |
| Fam214a | Trib2 | Ppox | Rnf19b | BC035044 | |
| Gm15518 | Mcoln3 | Prodh | Rnls | Asf1b | |
| Lrrc28 | Aven | Wipf1 | Rad18 | Serpinf1 | |
| Zfp982 | Hspbp1 | Tmem161a | Zfp563 | Hpgds | |
| Plpbp | Phxr2 | Cdc34b | Rgs12 | Rbfox2 | |
| Bag4 | Rev1 | A930019D19 | Ankrd26 | Notch3 | |
| Klk1b22 | Il22ra2 | N4bp2 | Mrps26 | Prrg4 | |
| Zfp954 | Zfp951 | Gm16568 | Pcnx4 | Ints5-1 | |
| Wdr43 | Muc13 | H2-T3 | Rcn1 | Tsfm | |
